# Therapeutic Potential of Antimicrobial Peptide PN5 against Multidrug-Resistant E. coli and Anti-Inflammatory Activity in a Septic Mouse Model

**DOI:** 10.1128/spectrum.01494-22

**Published:** 2022-09-21

**Authors:** Da Dam Kang, Jonggwan Park, Yoonkyung Park

**Affiliations:** a Department of Biomedical Science, Chosun University, Gwangju, South Korea; b Department of Bioinformatics, Kongju National University, Kongju, South Korea; c Research Center for Proteinaceous Materials (RCPM), Chosun University, Gwangju, South Korea; Riverside University Health System, Medical Center—University of California

**Keywords:** antimicrobial peptide, multidrug-resistant *E. coli*, septic shock, anti-inflammatory activity, lipopolysaccharide

## Abstract

Antibiotic-resistant bacteria have become a public health problem. Thus, antimicrobial peptides (AMPs) have been evaluated as substitutes for antibiotics. Herein, we investigated PN5 derived from Pinus densiflora (pine needle). PN5 exhibited antimicrobial activity without causing cytotoxic effects. Based on these results, we examined the mode of action of PN5 against Gram-negative and -positive bacteria. PN5 exhibited membrane permeabilization ability, had antimicrobial stability in the presence of elastase, a proteolytic enzyme, and did not induce resistance in bacteria. Bacterial lipopolysaccharide (LPS) induces an inflammatory response in RAW 264.7 macrophages. PN5 suppressed proinflammatory cytokines mediated by NF-κB and mitogen-activated protein kinase signaling. In C57BL/6J mice treated with LPS and d-galactosamine, PN5 exhibited anti-inflammatory activity in inflamed mouse livers. Our results indicate that PN5 has antimicrobial and anti-inflammatory activities and thus may be useful as an antimicrobial agent to treat septic shock caused by multidrug-resistant (MDR) Escherichia coli without causing further resistance.

**IMPORTANCE** Antibiotic-resistant bacteria are a global health concern. There is no effective treatment for antibiotic-resistant bacteria, and new alternatives are being suggested. The present study found antibacterial and anti-inflammatory activities of PN5 derived from Pinus densiflora (pine needle), and further investigated the therapeutic effect in a mouse septic model. As a mechanism of antibacterial activity, PN5 exhibited the membrane permeabilization ability of the toroidal model, and treated strains did not develop drug resistance during serial passages. PN5 showed immunomodulatory properties of neutralizing LPS in a mouse septic model. These results indicate that PN5 could be a new and promising therapeutic agent for bacterial infectious disease caused by antibiotic-resistant strains.

## INTRODUCTION

Antibiotics have been used to combat life-threatening infections and improve the health of many infected patients (1). However, antibiotic resistance of bacteria to a variety of conventional antibiotics has become one of the most important public health concerns worldwide. Countries in the Organization for Economic Cooperation and Development estimate that the cumulative economic losses from antibiotic resistance will reach $20 and 35 trillion U.S. dollars (USD) in 2050 (2, 3). Therefore, increases in antibiotic resistance are a serious concern for global public health.

Particularly, Escherichia coli is a pathogen responsible for bloodstream infections (4). Treatment of E. coli infection is difficult because this bacterium can form biofilms that are resistant to antibiotics (5). Moreover, E. coli is a global issue because various strains have developed resistance to conventional antibiotics (6). Since the 1980s, isolates of E. coli that produce extended-spectrum β-lactamases (ESBLs) have been detected worldwide (7). ESBLs are enzymes that hydrolyze the β-lactam ring of antibiotics such as penicillin and cephalosporin (8). The spread of antibiotic-resistant ESBL-producing bacterial strains led to increased use of carbapenems as antimicrobial agents to treat patients infected by ESBL-producing *Enterobacteriaceae* (9, 10). However, carbapenem-resistant strains have emerged, making treatment of this pathogen difficult (11). The World Health Organization has described carbapenem-resistant-*Enterobacteriaceae* as a critical priority for the development of antibiotics (12).

To overcome this problem, antimicrobial peptides (AMPs) have emerged as substitutes to antibiotics. AMPs have been isolated and characterized from a wide range of animal, plant, and bacterial species and play important roles in the host defense system and innate immunity of all species (13). AMPs are 12 to 50 amino acids in length and contain two or more positively charged residues, such as arginine, lysine, or histidine, and hydrophobic residues. They contain hydrophilic amino acid residues arrayed on one side and hydrophobic amino acid residues arrayed on the opposite side of the helical molecule. This amphipathicity of antimicrobial peptides allows them to disrupt the bacterial membrane lipid bilayer (14). AMPs exhibit numerous antimicrobial activities ranging from membrane permeabilization to acting on a range of cytoplasmic targets. Their amino acid composition, amphipathicity, cationic charge, and size allow them to attach to and insert into membrane bilayers to form pores through “barrel-stave,” “carpet,” or “toroidal-pore” mechanisms (15, 16). The cytoplasmic membrane is a frequent target of AMPs, but these peptides may also interfere with DNA and protein synthesis, protein folding, and cell wall synthesis (17). AMPs exhibit high affinities for the lipopolysaccharide (LPS) present in the outer membrane of Gram-negative bacteria (18). LPS serves as a physical barrier to protect bacteria from their surroundings. LPS is also recognized by the immune system as a marker of bacterial pathogen invasion and is responsible for development of the inflammatory response and, in extreme cases, endotoxic shock (19). The biophysical properties of AMPs and their mode of interaction with LPS determine their biological function, susceptibility of bacteria, and ability of LPS to activate the immune system.

We previously identified a novel antimicrobial peptide, PN5, isolated from a pine needle (PN) of Pinus densiflora. Four active peptides purified from PN showed antimicrobial activity, and PN5 (FKFLARTGKFL) was sequenced by Edman degradation (20).

In this study, we investigated the antimicrobial activity of PN5 against Gram-negative and Gram-positive bacteria, including multidrug-resistant (MDR) E. coli. We then confirmed that PN5 inhibited biofilm formation by carbapenem-resistant E. coli and Staphylococcus aureus. Moreover, PN5 was not cytotoxic toward mouse red blood cells (mRBCs) and RAW 264.7 cells, a mouse macrophage cell line. The mechanism of action of PN5 against E. coli and S. aureus was examined through membrane-related experiments. We evaluated the ability of E. coli to develop resistance to PN5 and antibiotics and the binding affinity of PN5 to E. coli LPS. Additionally, the anti-inflammatory effect of PN5 was examined, and NF-κB and mitogen-activated protein kinase (MAPK) signals were stimulated with E. coli LPS in RAW 264.7 cells. Additionally, we examined the *in vivo* therapeutic potential of PN5 in a murine model of septic shock caused by LPS and d-galactosamine (d-GalN) by measuring the survival rate and levels of proinflammatory cytokines and morphological changes in liver tissues by staining with hematoxylin and eosin. These results suggest that PN5 can be used as an antimicrobial and anti-inflammatory agent.

## RESULTS

### Characterization and structure analysis of PN5.

**FIG 1:**
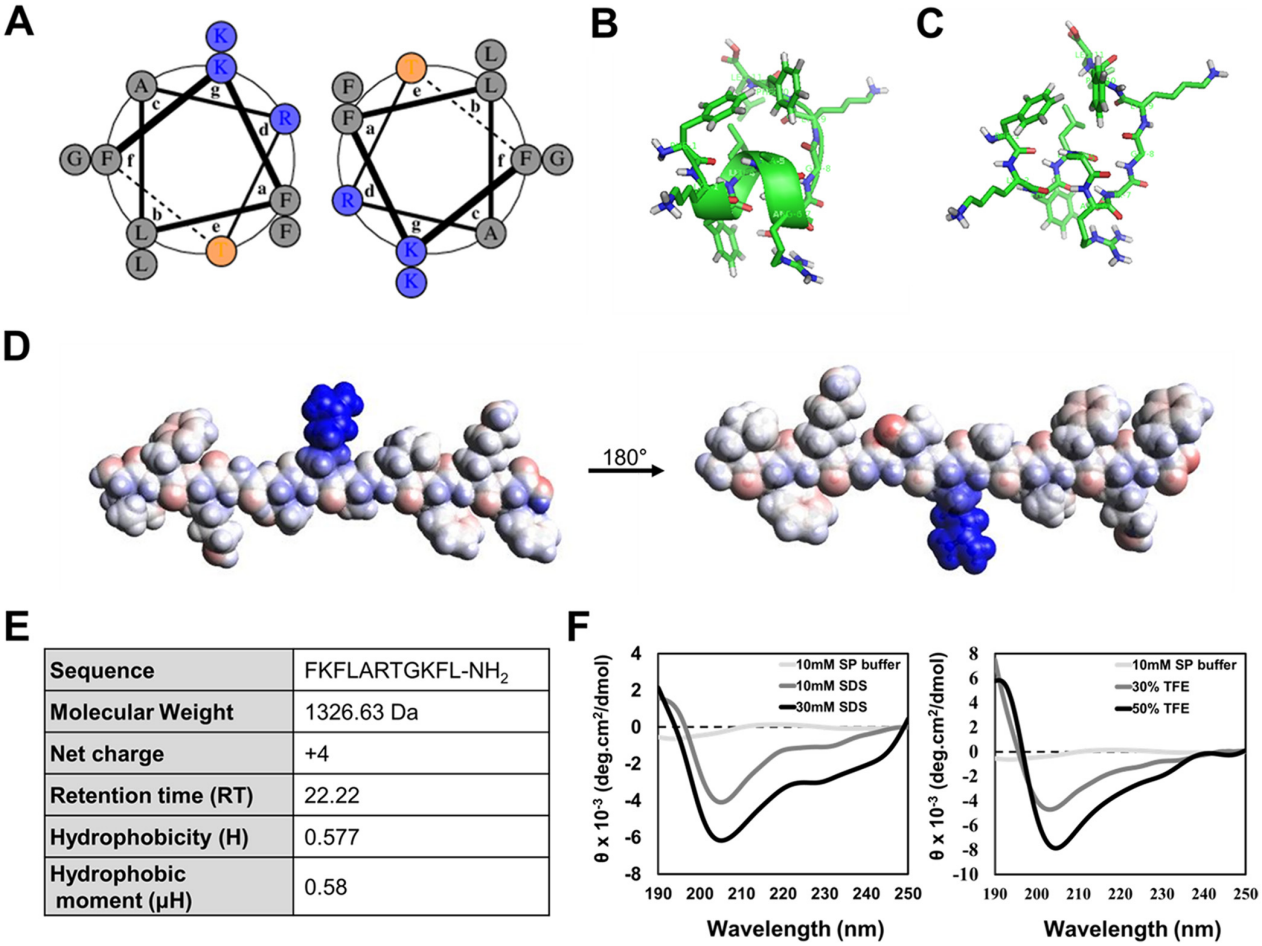
(A) Helical wheel diagram of PN5. Two chains are specified, assuming a homodimer. (B) 3D conformation of PN5 using PEPFOLD and PyMOL. Carbon, green; hydrogen, white; nitrogen, blue, oxygen, red; sulfur, yellow. (C) Stick and ball model of PN5 using PEPFOLD. (D) Electrostatic potentials were mapped onto PN5 surfaces. Areas with positive charges are shown in blue, negative charges in red, and hydrophobic residues in white. (E) Characteristics of PN5; (F) secondary protein structural analysis of PN5. Shown are results from CD spectroscopy analysis of PN5 in 10 mM sodium phosphate (SP) buffer with 10 mM and 30 mM sodium dodecyl sulfate (SDS) and 30% and 50% trifluoroethanol (TFE). The peptide concentration was fixed at 50 μM.

Helical wheel diagrams and three-dimensional structure projections were displayed to investigate structural determinants of PN5 activity. PN5 displays an α-helical structure in protein structure modeling (Fig. 1A to C). An electrostatic potential surface map was generated to display the charge distribution (Fig. 1D). Lysine and arginine residues of PN5 contributed to the positive charge (area in blue) on the peptide surface. The sequence, molecular weight, net charge, hydrophobicity, and hydrophobic moment of PN5 are listed in Fig. 1E. PN5 consists of 11 amino acids with a net charge, hydrophobicity, and hydrophobic moment of +4, 0.577, and 0.58, respectively. In reverse-phase high-performance liquid chromatography (RP-HPLC) on a C_18_ column, the retention time of PN5 was 22.22 min, and its molecular weight as verified by matrix-assisted laser desorption ionization–time of flight mass spectrometry was 1,326.6 Da (see Fig. S1A and B in the supplemental material).

The secondary structure of PN5 was estimated using circular dichroism (CD) spectroscopy in sodium dodecyl sulfate (SDS), which mimicked the negative charge of the bacterial membrane, and trifluoroethanol (TFE), which mimicked the hydrophobic environment of the bacterial membrane at various concentrations. PN5 showed a random coil having a negative minimum between 190 and 200 nm in 10 mM sodium phosphate buffer, which was used to mimic an aqueous environment. However, PN5 displayed an α-helical structure having a negative minimum between 208 and 222 nm in bacterial membrane-mimicking environments with increasing SDS and TFE concentrations (Fig. 1F). The estimated extent of helicity (percentage) of PN5 is presented in Table S1. The helicity of PN5 increased to 9.99% and 9.38% in 30 mM SDS and 50% TFE, respectively. The CD spectra of PN5 gradually shifted in concentration dependent on SDS and TFE. These results suggest that PN5 can form an α-helical structure in the bacterial membrane through electrostatic and hydrophobic interactions.

### Antibacterial activity of PN5.

**FIG 2:**
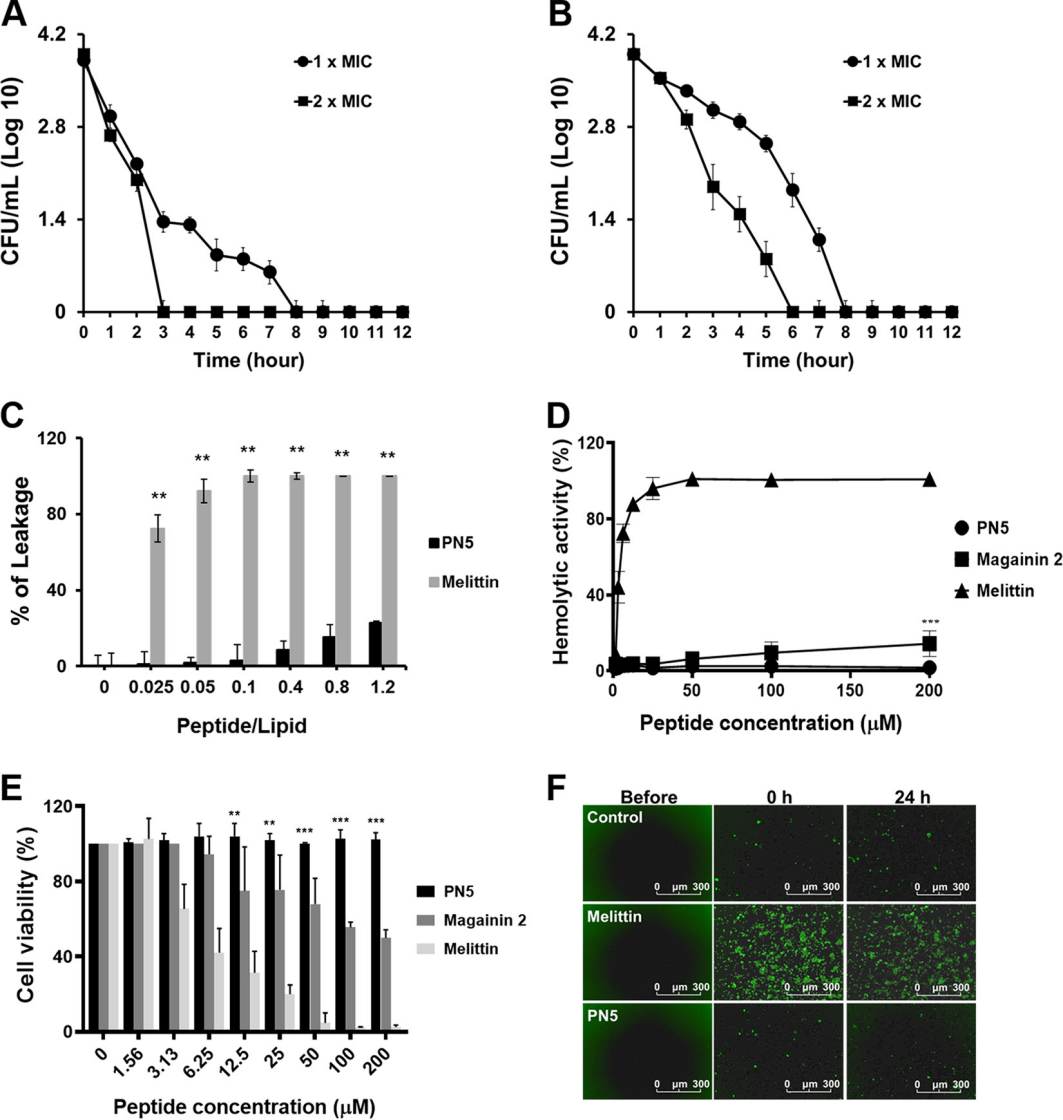
Measurements of toxicity of PN5. (A) E. coli (ATCC 25922) and (B) S. aureus (ATCC 25923) were incubated with PN5 at 1× and 2× MIC values. (C) Peptide-induced calcein leakage from artificial PC-CH-SM (1:1:1 [wt/wt]) large unilamellar vesicle (LUV) liposome. **, *P* < 0.01 versus control. (D) Hemolytic activity of peptides was measured against 8% mouse red blood cells. Values represent the mean ± standard error of the mean (SEM) from three individual experiments. (E) Viability of RAW 264.7 cells was assessed by MTT assay. (F) Real-time detection of cytotoxicity using the IncuCyte Live-Cell Imaging System and Cytotox green dye. RAW 264.7 cells were incubated for 24 h with 25 μM melittin and 200 μM PN5. **, *P* < 0.01, and ***, *P* < 0.001, versus melittin.

The antimicrobial activity of PN5 was investigated against Gram-negative strains of E. coli, Pseudomonas aeruginosa, Salmonella enterica serovar Typhimurium, and Acinetobacter baumannii and Gram-positive strains of S. aureus, Bacillus subtilis, and Listeria monocytogenes (Table 1 and Table S2). We used melittin found in honeybee venom and magainin 2 found in *Xenopus* skin as control peptides (21, 22). Both peptides have been reported to show antibacterial activity against Gram-negative and Gram-positive bacteria. PN5 displayed antimicrobial activity against both Gram-negative and Gram-positive bacteria, with MICs of 2 to 16 μM (2.65 to 21.22 μg mL^−1^). Magainin 2 showed activity against E. coli, P. aeruginosa, A. baumannii, S. aureus, and B. subtilis at concentrations of 8 to 64 μM (19.74 to 157.88 μg mL^−1^), but it showed no activity against *S.* Typhimurium and L. monocytogenes up to 64 μM (157.88 μg mL^−1^). Melittin at 2 to 4 μM (5.69 to 11.39 μg mL^−1^) displayed activity against all of the strains (Table S2). Moreover, PN5 and three antibiotics were investigated to determine antimicrobial activity against 19 strains of E. coli isolated from patients at Asan Hospital in South Korea (ASEC 1 to 19) (Table S3). Cefotaxime and ceftazidime mostly showed no activity at concentrations over 32 μM (14.58 and 17.49 μg mL^−1^, respectively) against the ASEC strains. Notably, meropenem did not exhibit antibacterial activity against ASEC 2, ASEC 3, and ASEC 4 up to 32 μM (12.27 μg mL^−1^). Magainin 2 did not show antimicrobial activity against E. coli ATCC 25922, ASEC 4, ASEC 17, and ASEC 19 up to 32 μM (78.94 μg mL^−1^). Melittin exhibited antimicrobial activity against ASEC from 2 μM to 8 μM (5.69 to 22.77 μg mL^−1^). PN5 showed antimicrobial activity against drug-resistant E. coli at 2 to 16 μM (2.65 to 21.22 μg mL^−1^) (Table 1). These results showed that PN5 had a higher antimicrobial activity than those of magainin 2, melittin, and the other antibiotics against clinical isolates. The antibacterial activity of PN5 was examined by measuring the time required to kill E. coli and S. aureus. CFU of E. coli were rapidly reduced within 3 h, and the colonies were completely killed after 8 h at 1× MIC of PN5. At 2× MIC of PN5, E. coli were completely killed within 3 h (Fig. 2A). S. aureus were completely killed within 8 h at a concentration of 1× MIC and after 6 h at a concentration of 2× MIC (Fig. 2B). These results indicate that PN5 was lethal against E. coli and S. aureus in a time- and dose-dependent manner.

**TABLE 1:** Antimicrobial activity of PN5 against E. coli ATCC 25922 and MDR Asan strains

Isolate^*a*^	MIC, μM (μg mL^−1^)^*b*^					
	PN5	Magainin 2	Melittin	Cefotaxime	Ceftazidime	Meropenem
ATCC 25922	16 (21.22)	64 (157.88)	2 (5.69)	<1 (<0.46)	<1 (<0.55)	<1 (<0.38)

ASEC 1	8 (10.61)	16 (39.47)	4 (11.39)	>32 (>14.58)	>32 (>17.49)	1 (0.38)
ASEC 2	2 (2.65)	8 (19.74)	4 (11.39)	>32 (>14.58)	>32 (>17.49)	>32 (>12.27)
ASEC 3	8 (10.61)	16 (39.47)	4 (11.39)	>32 (>14.58)	>32 (>17.49)	>32 (>12.27)
ASEC 4	4 (5.30)	>32 (>78.94)	8 (22.77)	>32 (>14.58)	>32 (>17.49)	>32 (>12.27)
ASEC 5	8 (10.61)	16 (39.47)	2 (5.69)	>32 (>14.58)	>32 (>17.49)	8 (3.07)
ASEC 6	4 (5.30)	4 (9.87)	4 (11.39)	>32 (>14.58)	>32 (>17.49)	16 (6.13)
ASEC 7	8 (10.61)	8 (19.74)	4 (11.39)	>32 (>14.58)	16 (8.75)	<1 (<0.38)
ASEC 8	4 (5.30)	16 (39.47)	4 (11.39)	>32 (>14.58)	>32 (>17.49)	<1 (<0.38)
ASEC 9	16 (21.22)	8 (19.74)	4 (11.39)	>32 (>14.58)	>32 (>17.49)	<1 (<0.38)
ASEC 10	8 (10.61)	16 (39.47)	4 (11.39)	>32 (>14.58)	32 (17.49)	<1 (<0.38)
ASEC 11	2 (2.65)	16 (39.47)	4 (11.39)	>32 (>14.58)	>32 (>17.49)	<1 (<0.38)
ASEC 12	2 (2.65)	16 (39.47)	4 (11.39)	>32 (>14.58)	>32 (>17.49)	16 (6.13)
ASEC 13	8 (10.61)	16 (39.47)	4 (11.39)	>32 (>14.58)	>32 (>17.49)	2 (0.77)
ASEC 14	2 (2.65)	4 (9.87)	2 (5.69)	>32 (>14.58)	>32 (>17.49)	8 (3.07)
ASEC 15	16 (21.22)	16 (39.47)	4 (11.39)	>32 (>14.58)	>32 (>17.49)	<1 (<0.38)
ASEC 16	4 (5.30)	8 (19.74)	4 (11.39)	>32 (>14.58)	>32 (>17.49)	<1 (<0.38)
ASEC 17	8 (10.61)	>32 (>78.94)	8 (22.77)	>32 (>14.58)	>32 (>17.49)	<1 (<0.38)
ASEC 18	16 (21.22)	16 (39.47)	4 (11.39)	>32 (>14.58)	>32 (>17.49)	<1 (<0.38)
ASEC 19	8 (10.61)	>32 (>78.94)	8 (22.77)	>32 (>14.58)	>32 (>17.49)	8 (3.07)

a Clinical isolates of multidrug-resistant (MDR) E. coli strains were obtained from Asan Hospital, South Korea.

b The minimum inhibitory concentration (MIC) was determined in three independent experiments.

### Cytotoxic and hemolytic activities of PN5.

First, we investigated the membrane permeabilization effect of PN5 and melittin by measuring the release of calcein entrapped within l-α-phosphatidylcholine (PC)-cholesterol (CH)-sphingomyelin (SM) (1:1:1 [wt/wt]) large unilamellar vesicles that mimic a eukaryotic membrane. At a peptide/lipid (P/L) ratio of 0.1, melittin, a positive control for membrane permeabilization, induced ~100% calcein leakage. However, PN5 induced less than 22.7% calcein release at a ratio of 1.2 (Fig. 2C). Moreover, we investigated hemolytic activity by measuring the release of hemoglobin from RBCs after incubation with peptides. Melittin induced 44.02% and 77.24% hemolysis at 3.125 and 6.25 μM and 100% hemolysis at 50 μM. In contrast, PN5 did not induce hemolysis even at a concentration of 200 μM (Fig. 2D). We further examined the toxicity of the peptide using RAW 264.7 cells derived from mouse macrophages by performing an MTT [3-(4,5-dimethyl-2-thiazolyl)-2,5-diphenyl-2H-tetrazolium bromide) assay. PN5 did not show toxicity toward RAW 264.7 cells at up to 200 μM. However, melittin and magainin 2 caused over 50% toxicity at 6.25 and 200 μM (Fig. 2E). To visualize cell survival after exposure to PN5 and melittin, we used Cytotox green, which is suitable for visualizing cell death. PN5 at 200 μM caused no toxicity for 24 h, as demonstrated by the lack of increase in fluorescence intensity, whereas melittin immediately damaged the cells (Fig. 2F). These results suggested that PN5 exhibited no cytotoxicity.

### Antibiofilm activity of PN5.

**FIG 3:**
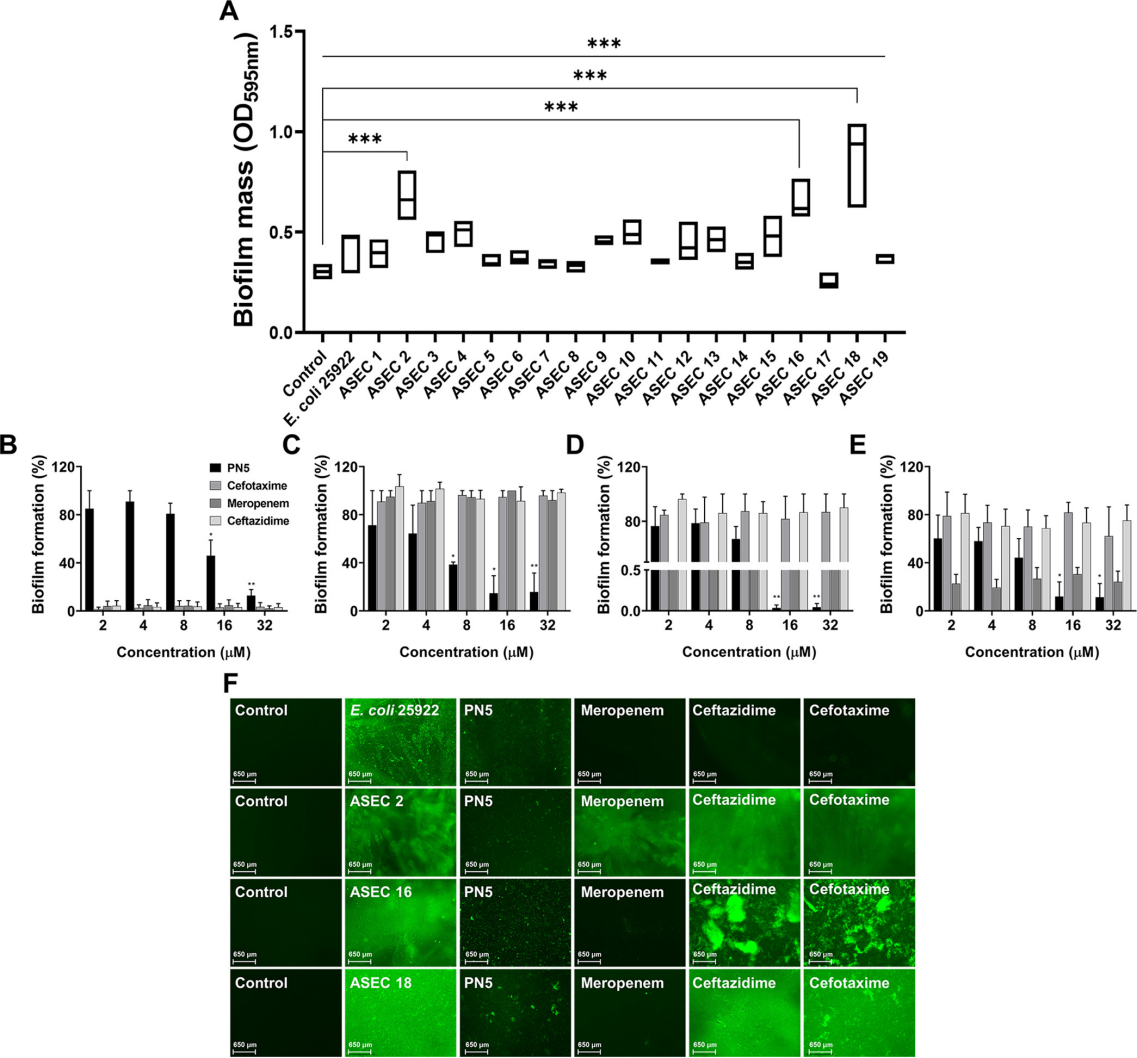
Biofilm-forming ability of E. coli (ATCC 25922) and clinical isolates. (A) E. coli (ATCC 25922) and ASEC 1 to 19 strains were seeded at 5 × 10^5^ CFU mL^−1^ to form biofilms. A 10 mM concentration of SP buffer-treated (negative control) and the dyed biofilm was measured at 595 nm. ***, *P* < 0.001 versus control by unpaired *t* test. (B) Biofilm formation of E. coli (ATCC 25922) was measured by treatment with PN5 and antibiotics at different concentrations. ASEC 2 (C), ASEC 16 (D), and ASEC 18 (E) were treated with PN5 and antibiotics to measure the inhibition of biofilm formation. (F) Formed biofilm stained with fluorescent SYTO 9 was visualized. PN5 (16, 8, 16, and 8 μM, respectively) and antibiotics (32 μM) were used to treat E. coli (ATCC 25922), ASEC 2, ASEC 16, and ASEC 18. The biofilms stained with SYTO 9 were visualized using the EVOS FL color imaging system. Values represent the mean ± SEM of values from three individual experiments. *, *P* < 0.02; **, *P* < 0.01; and ***, *P* < 0.001, by unpaired *t* test.

Biofilm is an extracellular polymer matrix in which single bacteria form colonies and attach to surfaces. Biofilms formed by bacteria can become resistant to antibiotics (23). E. coli ATCC 25922, S. aureus ATCC 25923, and three MDR E. coli strains (ASEC 2, 16, and 18) that effectively form biofilms were used to determine whether PN5 inhibits biofilm formation (Fig. 3A and Fig. S2). As shown in Table 2, PN5 inhibited biofilm formation by the strains of E. coli ATCC 25922, ASEC 2, ASEC 16, and ASEC 18 by more than 50% at 8 and 16 μM (10.61 and 21.22 μg mL^−1^). However, meropenem, ceftazidime, and cefotaxime did not inhibit biofilm formation by ASEC. PN5 significantly inhibited the biofilm formation by E. coli ATCC 25922, ASEC 2, ASEC 16, and ASEC 18 (Fig. 3B to E). Cefotaxime and ceftazidime did not inhibit biofilm formed by ASEC 16 and 18 at ≥32 μM (≥69.95 and 58.3 μg mL^−1^, respectively). Meropenem inhibited biofilm formation by ASEC 16 and 18 but did not show antibiofilm activity against ASEC 2. We also treated the cells with PN5 followed by cell staining with live BacLight staining SYTO9 to visualize biofilm formation PN5. The biofilm formation significantly reduced in the presence of PN5. However, meropenem, ceftazidime, and cefotaxime did not inhibit biofilm formation even at 32 μM (14.58, 17.49, and 12.27 μg mL^−1^, respectively) against ASEC 2 (Fig. 3F). These results suggest that PN5 has antibiofilm activity against E. coli.

**TABLE 2:** MBIC_50_ of PN5 and antibiotics against E. coli ATCC 25922, ASEC 2, ASEC 16, and ASEC 18

Isolate	MBIC_50_, μM (μg mL^−1^)^*a*^			
	PN5	Ceftazidime	Cefotaxime	Meropenem
ATCC 25922	16 (21.22)			

ASEC 2	8 (10.61)	>128 (>69.95)	>128 (>58.3)	64 (24.54)
ASEC 16	16 (21.22)	>128 (>69.95)	128 (58.3)	<2 (<0.77)
ASEC 18	16 (21.22)	>128 (>69.95)	>128 (>58.3)	<2 (<0.77)

a MBIC_50_, minimum inhibitory concentration resulting in at least 50% inhibition of biofilm formation.

### Permeability of PN5 to bacterial outer and inner membranes.

**FIG 4:**
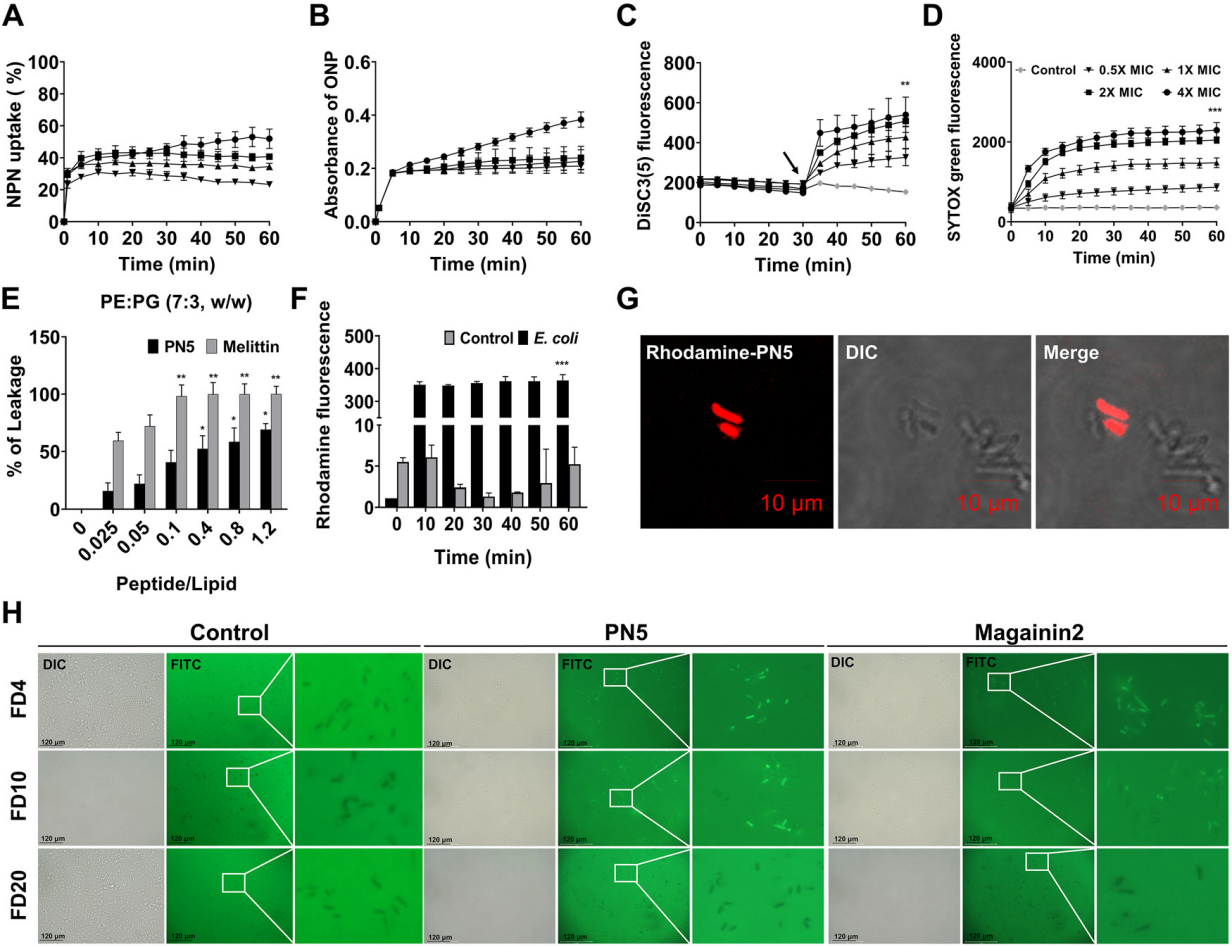
Verification of PN5 action against bacterial membrane. (A) Outer membrane permeability observed as NPN uptake. E. coli (ATCC 25922) was incubated with NPN in the presence of 0.5×, 1×, 2×, and 4× MIC of PN5. (B) The *o*-nitrophenyl-β-galactosidase (ONPG) assay was performed to evaluate the inner membrane permeability of PN5 against E. coli ATCC 25922. The absorbance of the hydrolysate was measured at 420 nm. (C) Various indicated concentrations of PN5 were added at 30 min (marked with an arrow) to E. coli ATCC 25922 along with DiSC_3_(5). Cytoplasmic membrane potential changes were observed at 622 nm (excitation) and 670 nm (emission). (D) Fluorescence intensity of SYTOX green was measured at 485 nm (excitation) and 520 nm (emission). (E) Calcein leakage from artificial PE-PG (7:3 [wt/wt]) liposomes in the presence of PN5 and melittin. (F) Rhodamine-labeled PN5 was used to treat E. coli ATCC 25922. PN5 labeled with rhodamine (2× MIC) was added, and fluorescence was observed using a fluorescence spectrophotometer for 60 min. (G) Localization of rhodamine-labeled PN5 on E. coli (ATCC 25922) was observed using confocal laser scanning microscopy. Scale bar of E. coli = 10 μm. (H) Fluorescein isothiocyanate (FITC)-dextran (FD) 4, 10, and 20 were treated with 2× MIC of peptides on E. coli ATCC 25922. Fluorescence imaging was performed using an EVOS cell imaging system: (i) differential interference contrast, (ii) FITC, and (iii) extended version, Scale bars = 120 μm. All values represent the mean ± SEM from three individual experiments. **, *P* < 0.01, and ***, *P* < 0.001, versus control.

We next examined the effect of PN5 on the bacterial cell membranes. An *N*-phenyl-1-naphthylamine (NPN) uptake assay was performed to confirm that the peptides disrupted the outer membrane of E. coli. Incubation with PN5 at 4× MIC for 60 min increased the fluorescence intensity by 47.6% (Fig. 4A). Following treatment magainin 2, NPN uptake was 35.7% at 4× MIC in 60 min. After treatment of the cells with melittin, NPN uptake was 82.8% at 4× MIC (Fig. S3). The permeability of the inner membrane by PN5 was further examined in a 2-nitrophenyl-β-d-galactopyranoside (ONPG) assay. E. coli has a native lactose operon that contains a gene that encodes LacZ. First, ONPG passes through the inner membrane to access cytoplasmic LacZ. The β-galactosidase enzyme LacZ hydrolyzes ONPG to *o*-nitrophenol (ONP), causing a yellow coloration. Treatment with PN5 dose-dependently induced a color change at concentrations lower than those for the change induced by melittin (Fig. 4B and Fig. S4A). Moreover, we investigated the effect of peptides on the membrane potential of the bacterial cytoplasmic membranes using 3,3′-dipropylthiacarbocyanine iodide [DiSC_3_(5)]. DiSC_3_(5) accumulates in the cytoplasmic membrane for 30 min. However, damage to the cytoplasmic membrane induces release of the dye, which increases the fluorescence intensity. PN5 and magainin 2 induced similar increases in fluorescence intensity in a dose-dependent manner in E. coli. Melittin increased the fluorescence intensity more than that of both peptides (Fig. 4C and Fig. S4B). SYTOX green uptake was conducted to investigate the integrity of the bacterial membrane after exposure to the peptides (Fig. 4D and Fig. S5). SYTOX green cannot cross intact bacterial membranes. However, if the bacterial membrane is compromised, the dye binds to DNA, causing an increase in fluorescence intensity. PN5 increased SYTOX green uptake by E. coli in a dose-dependent manner (Fig. 4D). Calcein leakage from phosphatidylethanolamine (PE)-phosphatidylglycerol (PG) (7:3 [wt/wt]) liposomes, which mimic the Gram-negative bacterial membrane, was evaluated to determine whether PN5 perturbs artificial liposomes. Calcein leakage of 69.2% was caused by PN5 at a P/L (peptide/lipid) ratio of 1.2. Melittin caused ~100% of the calcein to be released at the P/L ratio of 0.1 in liposomes, whereas calcein leakage was induced by PN5 below 70% (Fig. 4E). To examine this visually, rhodamine-labeled PN5 was added to E. coli and its surface localization was observed. Rhodamine-labeled PN5 was localized at the cell surface and exhibited red fluorescence in linear E. coli. This indicates that rhodamine-labeled PN5 was bound to the bacterial membranes of E. coli (Fig. 4F and G). We evaluated the membrane permeabilization effect of PN5 against S. aureus, and PN5 exhibited an ~1.5-fold-higher permeabilization effect on E. coli than that on S. aureus (Fig. S6). To evaluate the size of the pores formed by PN5 in E. coli, the efflux of different-sized fluorescein isothiocyanate (FITC)-dextran (FD) was visualized using a fluorescence microscope (Fig. 4H). PN5 exhibited intrinsic fluorescence in E. coli treated with FD 4 and FD 10, as in magainin 2. Magainin 2 is an AMP that typically has functions through a toroidal-pore mechanisms. The data clearly demonstrated that PN5 forms pores of 2.8 to 4.6 nm in the bacterial membrane through a toroidal-pore mechanism, similar to that of magainin 2.

### Induction of drug resistance.

**FIG 5:**
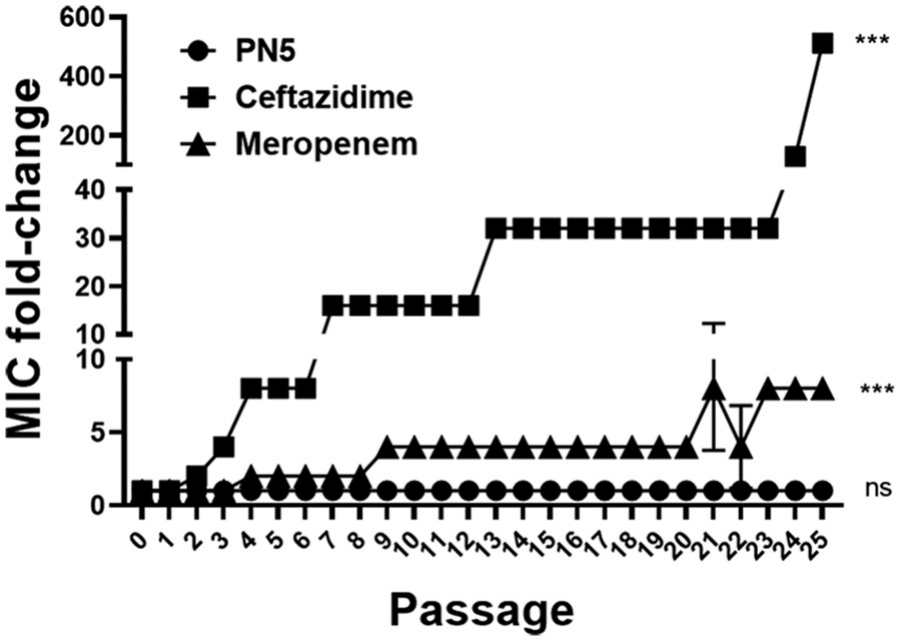
Antimicrobial activity was measured up to 25 passages by treating 0.5× MIC of PN5, ceftazidime, and meropenem against E. coli (ATCC 25922). Passaging was performed at 2 × 10^5^ CFU mL^−1^ of E. coli cultured on the previous day, and the fold change in MIC represents the ratio of the MIC after each passage to the initial MIC before the first passage. Values represent the mean ± SEM from three individual experiments. Values represent the mean. ns, not significant, and ***, *P* < 0.001, versus control by unpaired *t* test.

To verify that PN5 has a low risk for resistance development, E. coli ATCC 25922 cells up to passage 25 (the step number of the passage) were evaluated. At passage 1, ceftazidime and meropenem showed MIC values of 0.25 and 0.03125 μM against E. coli. However, after 25 passages, ceftazidime increased the MIC by 512-fold to 128 μM and meropenem increased the MIC by 8-fold to 0.25 μM (Table S4). In contrast, PN5 retained its MIC value at 16 μM. These results suggest that E. coli does not develop resistance to PN5 (Fig. 5).

### Resistance of PN5 against proteolytic degradation.

**FIG 6:**
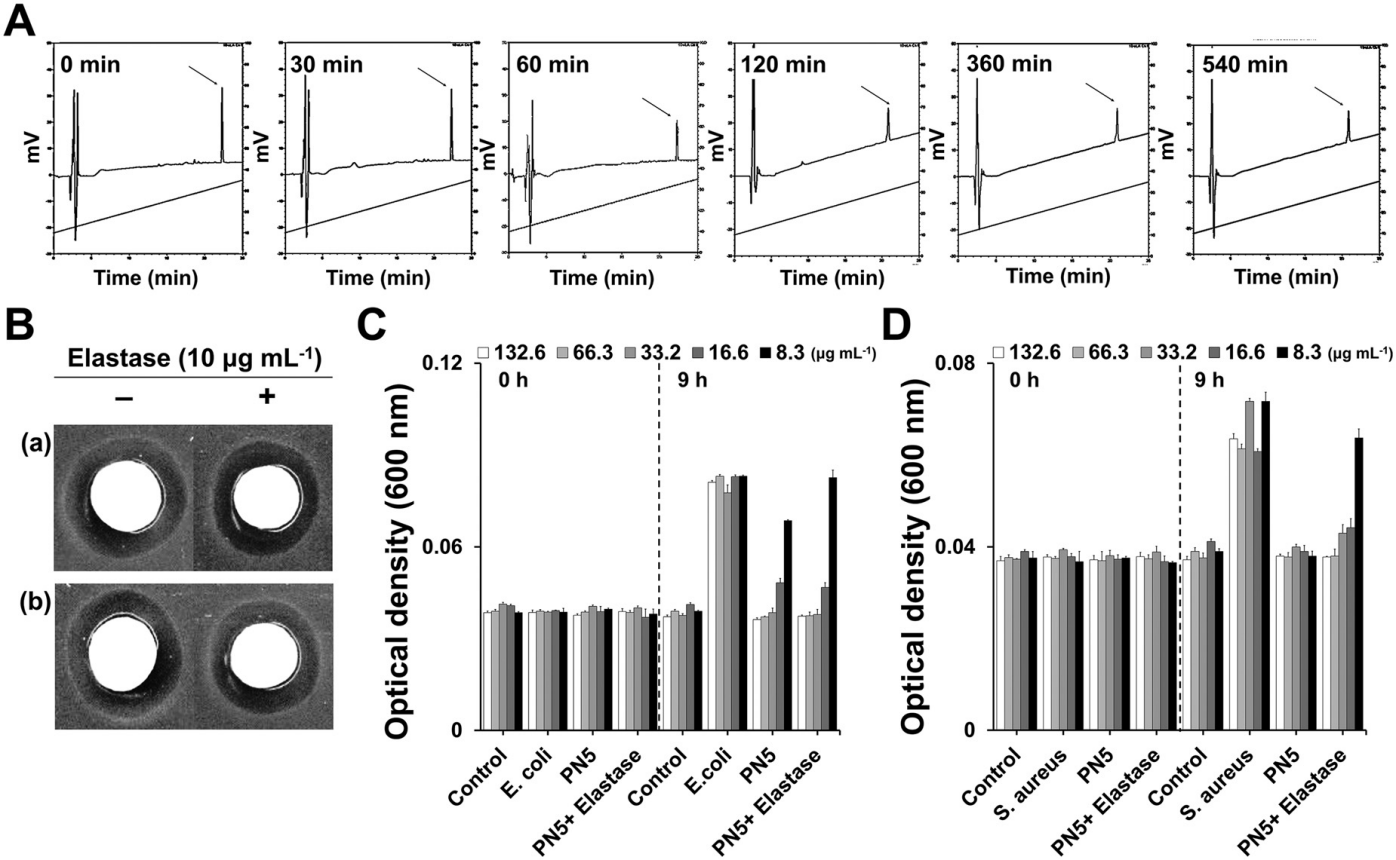
Evaluation of enzyme stability and antimicrobial activity of PN5. (A) Enzyme stability determined using RP-HPLC (C_18_ column; detection wavelength = 214 nm). PN5 (50 μg) was treated with 10 μg mL^−1^ elastase in 0.1 M Tris-HCl buffer (pH 8.0) at different time points at 37°C. *x* axis, time; *y* axis, absorbance measured at 214 nm. (B) Disc assays to determine antimicrobial activity with 50 μg PN5 and 50 μg PN5 pretreated with 10 μg mL^−1^ elastase for 9 h. (B to D) (a) E. coli (ATCC 25922) and (b) S. aureus (ATCC 25923), (C) E. coli (ATCC 25922), and (D) S. aureus (ATCC 25923) were treated with different concentrations of PN5 and elastase-treated PN5 for 0 h and 9 h at 37°C. OD_600_ was determined using a microplate reader. All values represent the mean ± SEM from three individual experiments.

AMPs exhibit unstable bioactivity toward proteolytic enzymes. We evaluated the stability of PN5 in the presence of elastase, a member of the serine-protease family. Elastase is secreted during inflammation (24). PN5 was incubated with elastase for 0, 30, 60, 120, 360, and 540 min. After incubation of PN5 with elastase, the peak of PN5 was separated using RP-HPLC. The peak of residual PN5 remained even after 9 h (Fig. 6A), indicating that it was not strongly affected by the protease. Moreover, the susceptibility of the peptide treated with elastase against bacteria was assessed. The inhibition zones of PN5 and elastase-treated PN5 were determined against E. coli [Fig. 6B (a)] and S. aureus [Fig. 6B (b)]. Treatment with elastase did not affect the antimicrobial activity of PN5. PN5 as well as the combination of PN5 and elastase were used to treat the cells, and the degree of bacterial growth was compared by optical density (OD). At concentrations below 16.6 μg mL^−1^ (12.5 μM) of elastase-treated PN5, the bacteria propagated, although most bacteria did not grow at concentrations higher than 33.2 μg mL^−1^ (25 μM) (Fig. 6C and D). This suggests that PN5 is resistant to proteolytic degradation by elastase.

### Immunomodulatory activity of PN5 associated with interaction of bacterial outer membrane component.

**FIG 7:**
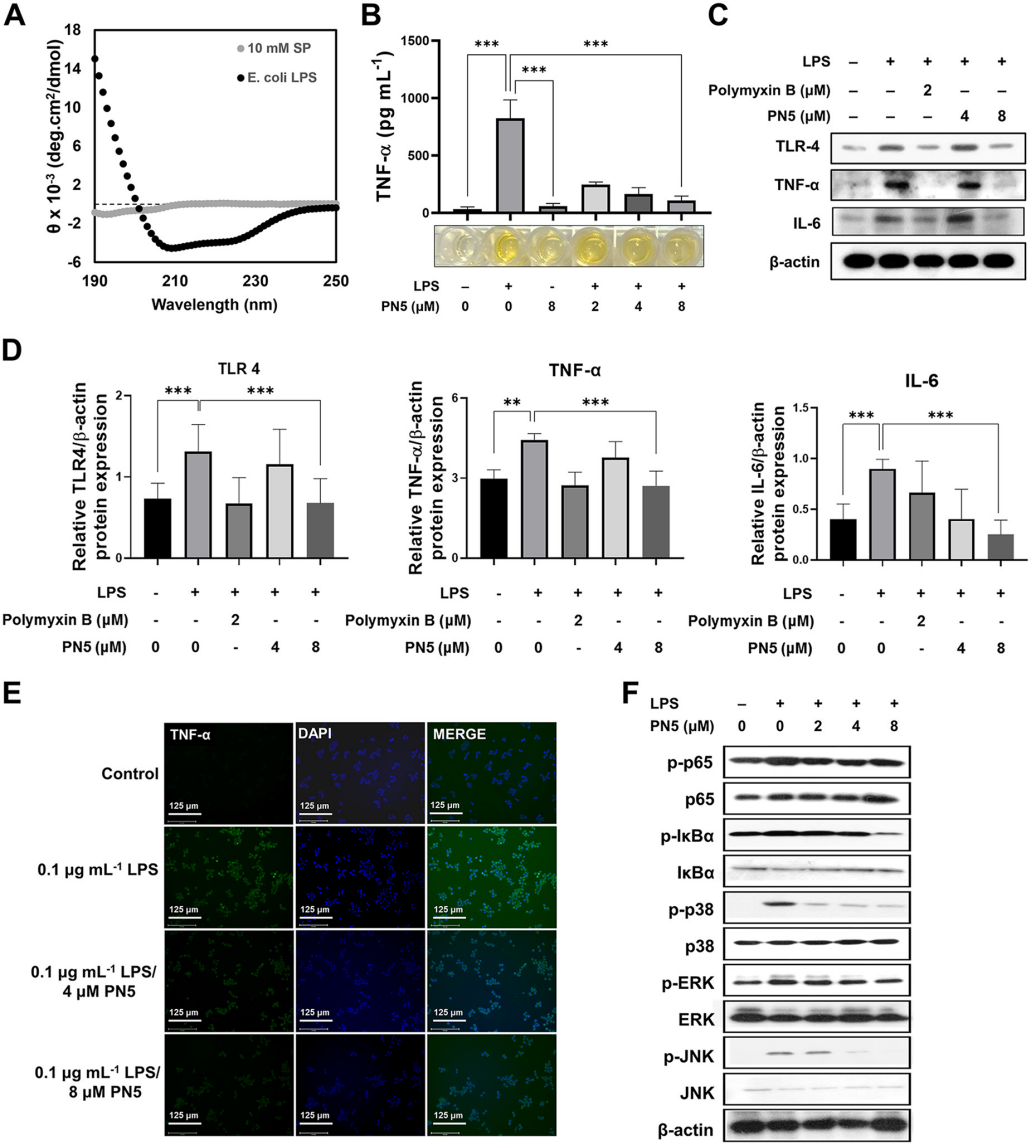
Interaction with E. coli O111:B4 lipopolysaccharide and immunomodulatory activity of PN5. (A) CD analysis of the binding structure of AMPs with lipopolysaccharide (LPS); (B) effect of PN5 (2, 4, and 8 μM) on LPS-induced TNF-α production. The concentrations of TNF-α in the culture supernatants were determined by ELISA. (C) Western blot of TNF-α, TLR4, and β-actin expression. LPS-pretreated RAW 264.7 cells were cultured with PN5 at different concentrations for 24 h. (D) Graphs were generated by quantifying the Western blots using ImageJ software. (E) Immunofluorescence staining showed that PN5 inhibited TNF-α expression induced by E. coli LPS. (F) The Western blot was performed for NF-κB and MAPK signaling activation in RAW264.7 cells. Intracellular NF-κB (p65), IκBα, p38, ERK, and JNK signals were confirmed. Values represent the mean ± SEM from three individual experiments. **, *P* < 0.01, and ***, *P* < 0.001, by unpaired *t* test.

AMPs must interact with LPS and lipoteichoic acid (LTA) in the Gram-negative and -positive bacterial membrane to exert their antimicrobial and anti-inflammatory activities (19). CD spectra were determined to examine the secondary structures of PN5 in the presence of LPS and LTA from E. coli and S. aureus. PN5 exhibited a random coil structure in 10 mM sodium phosphate buffer, but it changed from a random coil structure to an α-helix structure in the presence of LPS and LTA (Fig. 7A and Fig. S6A). The helicity of PN5 increased to 14.13% and 31.55% in LPS and LTA, respectively (Table S1). To assess the ability of PN5 to modulate inflammation, RAW 264.7 cells were treated with LPS and LTA. To investigate the anti-inflammatory activity of PN5, the levels of proinflammatory cytokines such as tumor necrosis factor alpha (TNF-α), interleukin-6 (IL-6), and IL-1β in these cells were analyzed by reverse transcription-PCR (RT-PCR). LPS and LTA increased proinflammatory cytokine levels. However, PN5 showed inhibitory effects on the LPS- and LTA-induced production of TNF-α, IL-6, and IL-1β (Fig. S7). LPS-stimulated TNF-α expression was gradually suppressed by PN5 in RAW 264.7 cells. We performed an enzyme-linked immunosorbent assay (ELISA) to measure the levels of TNF-α, a proinflammatory cytokine. LPS induced 824.97 pg mL^−1^ of TNF-α, but the level was reduced to 108.47 pg mL^−1^ by PN5 at 8 μM (Fig. 7B). Polymyxin B has a high affinity for LPS, and it has an LPS neutralizing effect; therefore, it was used as a positive control (25). Thus, Western blotting showed that PN5 decreased Toll-like receptor 4 (TLR4), a proinflammatory cytokine with a key role in inducing immune responses, TNF-α and IL-6 levels (Fig. 7C and D). To visualize immunomodulatory activity of PN5, treatment with LPS increased TNF-α levels in RAW 264.7 cells; however, little staining was observed in infected cells treated with PN5 (Fig. 7E). We identified the intracellular signal via which PN5 regulated the expression of NF-κB (p65) and MAPK (Fig. 7F). The expression of TNF-α and p-NF-κB due to LPS stimulation showed the greatest increase at 24 h compared to the control (Fig. S8). At this time point, the expression of the phosphorylated forms of the various proteins (p-p65, p-IκBα, p-ERK, p-JNK, and p-p38) was downregulated after treatment with PN5 (Fig. 7F). These data suggest that PN5 has anti-inflammatory activity *in vitro*.

### *In vivo* efficacy of PN5 in E. coli LPS/d-GalN-induced septic shock mouse model.

**FIG 8:**
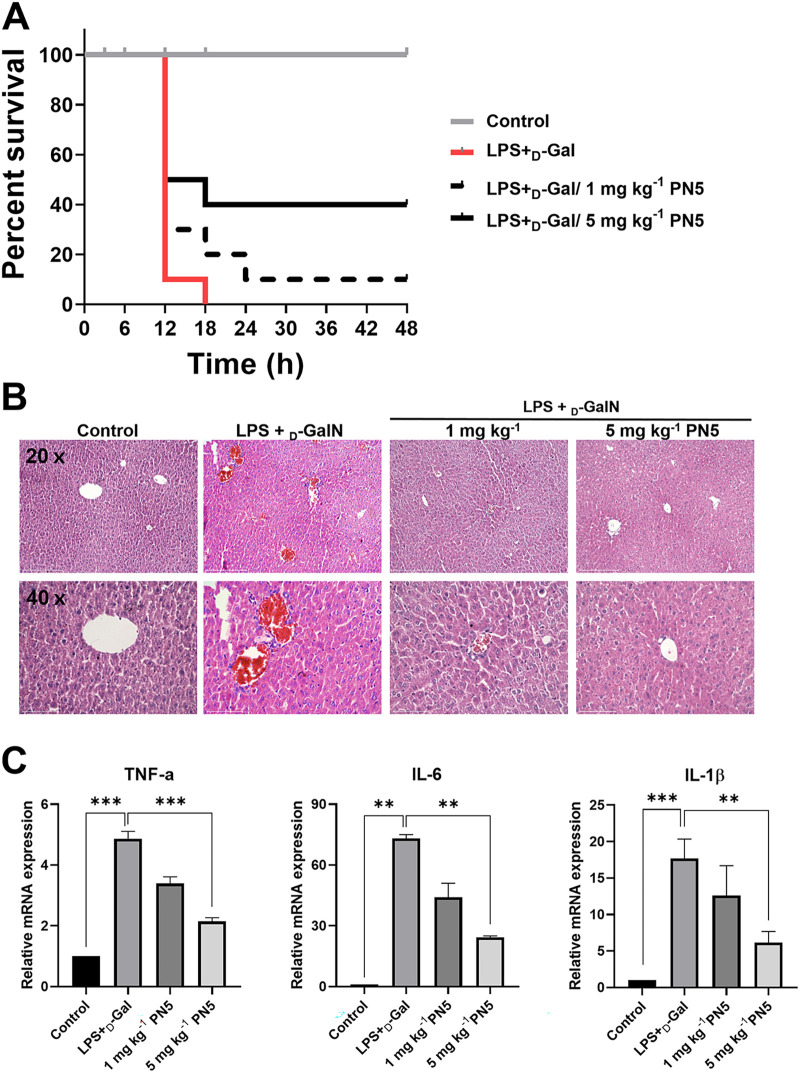
Effect of PN5 on model of *in vivo* endotoxic shock induced by LPS and d-galactosamine (d-GalN). (A) Survival rate of mice after LPS injection. At 30 min after intraperitoneal injection of LPS, 1 and 5 mg kg^−1^ of PN5 were intraperitoneally injected. The mortality rate of mice was observed until 48 h (all groups, *n* = 10). (B) Histology of liver tissues from C57BL/6J mice. PN5 (1 and 5 mg kg^−1^) was injected 30 min after treatment with 16 μg kg^−1^ LPS and 500 mg kg^−1^ d-GalN via intraperitoneal administration. Mouse liver sections were stained with hematoxylin and eosin. Thin sections of the liver tissues were observed at magnifications of 20× and 40× (all groups, *n* = 4). (C) Real-time PCR analysis of TNF-α, IL-6, and IL-1β cytokine mRNA in liver tissues of C57BL/6J mice. Mice were treated with LPS and d-GalN 30 min prior to induction of endotoxic shock. Liver tissues were harvested at 6 h after treatment with 1 and 5 mg kg^−1^ PN5. mRNA expression levels in infected animals were compared to those in uninfected controls. Values represent the mean ± SEM. **, *P* < 0.01, and ***, *P* < 0.001, by unpaired *t* test.

We induced septic shock in mice by treatment with E. coli LPS and d-galactosamine (d-GalN) to induce septic shock (26). We confirmed that liver was damaged (Fig. S9A and B). To evaluate the immunomodulatory effect of PN5 *in vivo*, 1 and 5 mg kg^−1^ PN5 were intraperitoneally injected in a mouse model treated with E. coli LPS and d-GalN (Fig. 8A). E. coli LPS- and d-GalN-induced septic shock killed all mice after 18 h. However, treatment with 1 and 5 mg kg^−1^ PN5 improved the survival rates to 10% and 40%, respectively, at 48 h. We confirmed that TNF-α, alanine aminotransferase (ALT), and aspartate aminotransferase (AST) levels significantly increased in LPS- and d-GalN-treated mice groups, and the increase aggravated the liver injury. However, TNF-α, AST, and ALT levels in the serum decreased after PN5 administration (Fig. S9C and D). Histopathological analysis revealed neutrophil recruitment and immense hemorrhaging in the livers of mice treated with E. coli LPS and d-GalN. PN5 administration reduced inflammatory symptoms in the mouse liver (Fig. 8B). Moreover, the mRNA expression of TNF-α, IL-6, and IL-1β (4.87-, 73.2-, and 17.7-fold changes compared to controls) was increased by E. coli LPS and d-GalN. However, after administration of PN5 at 5 mg kg^−1^, the levels of the proinflammatory cytokines TNF-α, IL-6, and IL-1β were reduced (4.87- to 2.15-fold changes, 73.2- to 24.2-fold changes, and 17.7- to 6.15-fold changes, respectively) (Fig. 8C). These results suggest that treatment with d-GalN and E. coli LPS sensitizes the immune system of mice, and administration of PN5 inhibits the inflammatory response caused by E. coli LPS and d-GalN *in vivo*.

## DISCUSSION

The rapid rise of antibiotic-resistant bacteria is a serious global public health problem for which new antibacterial agents must be developed. AMPs, which are vital elements of the innate immune system, are promising candidates for treating bacterial infections. AMPs represent a potential class of antibiotics as they possess a distinctive antimicrobial mechanism compared with traditional antibiotics (27).

Traditionally, plant extracts have been developed as botanical medicines to treat diseases (28). Needles, pollen, cones, and cortices derived from pine trees have been used as antibacterial, anti-inflammatory, and preservative agents in traditional medicine (29). Particularly, pine needles are crude drugs that are effective for preventing diseases such as rheumatism, gastroenteric trouble, and asthma (30, 31). PN5, which consists of 11 amino acids, was isolated from pine needles and showed antibacterial activity against foodborne bacteria in a previous study (20).

In this study, we confirmed that PN5 has antimicrobial activities against Gram-negative and Gram-positive bacteria, including MDR E. coli. The ASEC strains that were clinically isolated from patients, mentioned in Table S3 in the supplemental material, produced ESBL, which induces resistance to β-lactam antibiotics, and New Delhi metallo-β-lactamase (NDM), which hydrolyzes a class of carbapenems. Therefore, antibiotics such as cefotaxime, ceftazidime, and meropenem exhibited no antimicrobial activity against some MDR E. coli. The four PN peptides (PN5, PN7, PN8, and PN10) purified from pine needles caused less than 20% cytotoxicity at 200 μg/mL^−1^ according to a previous study (20). Correspondingly, PN5 showed no hemolytic activity or cytotoxicity against mRBCs and RAW 264.7 cells. Moreover, PN5 did not induce leakage of calcein from PC-CH-SM (1:1:1 [wt/wt]) large unilamellar vesicles, which are artificial liposomes that mimic eukaryotic membranes, compared to melittin. These results indicate that PN5 is a safe agent for treating bacterial infections.

AMPs adopt secondary structures such as α-helices, β-sheets, and loops (32). A previous study confirmed that PN5 has an α-helical amphipathic structure in the three-dimensional (3D) representation (20). We used CD spectroscopy to examine the secondary structure of PN5 in bacterial membrane environments. In SDS and TFE, which mimic negatively charged and hydrophobic bacterial membranes, respectively, PN5 displays an α-helical structure. Structurally, PN5 has amphipathicity composed of positively charged amino acids (arginine and lysine) and hydrophobic amino acids (phenylalanine) involved in electrostatic interactions with the bacterial membrane, which is negatively charged (33). The hydrophobicity of PN5 allows it to bind to hydrophobic phospholipids in the bacteria (Fig. S10A).

PN5 displays effective antibacterial activity against clinical isolates. Biofilms formed by bacteria are less sensitive to antimicrobial agents than planktonic cells because of their self-produced protective extracellular matrix (34). Therefore, therapeutic agents for infectious diseases should inhibit biofilm formation, which is an important regulator of growth and adhesion to surfaces. E. coli and S. aureus can easily form biofilms that increase their resistance to antimicrobial agents and host defense (35). They can lead to biofilm-related chronic infections, such as catheter infections, urinary tract infections, and lung infections (36). We found that PN5 inhibited the biofilm formed by E. coli ATCC 25922, S. aureus ATCC 25923, and MDR E. coli strains, whereas meropenem, cefotaxime, and ceftazidime did not exhibit inhibition effects. PN5 exhibited higher antimicrobial and antibiofilm activities against ASEC2, isolated from patients, than those against E. coli ATCC 25922. This finding indicated that ASEC 2 and E. coli ATCC 25922 have different genomes, resulting in varied responses to PN5 (37).

The mode of action of PN5 against Gram-negative and Gram-positive bacteria was evaluated and compared with those of magainin 2 and melittin. When the outer membrane is damaged, NPN binds to the hydrophobic region of the membrane and exhibits increased fluorescence. The permeability of PN5 to the outer membrane was similar to that of magainin 2 (Fig. 4A and Fig. S3). Subsequently, we examined the inner membrane permeabilization and membrane depolarization of PN5 in bacteria using ONPG and DiSC_3_(5). β-Galactosidase from the lactose operon system is found across the inner membrane of E. coli. The cationic dye DiSC_3_(5) was translocated to the cytoplasmic membrane, resulting in the self-quenching of fluorescence, whereas damage of the bacterial membrane causes potential release of the DiSC_3_(5), resulting in an increase of fluorescence intensity. The permeability the E. coli cytoplasmic membrane was significantly higher than that of the S. aureus cytoplasmic membrane (Fig. 4C and Fig. S6). PN5 showed a difference in membrane permeability between Gram-negative and Gram-positive bacteria, possibly because of differences in the cell wall structure between these bacteria. Rhodamine-labeled PN5 was localized to bacteria, and SYTOX green revealed the membrane-permeabilization activity of PN5 against E. coli. Furthermore, we hypothesized that PN5 used a similar membrane interaction model as magainin 2. In the toroidal-pore model, the helical region of peptide is inserted into the bacterial membrane and aligned by interacting with the head groups of lipids in the membrane (38). Typically, magainin 2 uses a toroidal-pore model to interact with bacterial membranes (15). Imura and Matsuzaki determined the size of pores formed by magainin 2 functioning through the toroidal model using FITC-dextran (FD) (39). Based on this, the size of pores formed by PN5 was also measured using FD, which showed that PN5 formed a pore size similar to the diameter of FD 10 (~4.6 nm) and as toroidal pores with lipid flip-flop to induce membrane permeabilization (40). Thus, PN5 exerts its antimicrobial activity via a toroidal-pore model.

In terms of their physiological function, AMPs also show several disadvantages (41). First, AMPs may show weak stability in the presence of enzymes that decompose amino acids. To evaluate stability in the presence of protease, PN5 was coincubated with elastase (Fig. 6). Elastase is actively secreted during inflammation and is widely used to evaluate inflammatory responses *in vivo* (42). The major peak of PN5 was maintained for 9 h. After incubation with elastase, E. coli and S. aureus were treated with PN5 to determine whether antimicrobial activity was maintained. Antimicrobial activity was observed for up to 9 h, in accordance with the results of the disk diffusion test. These findings demonstrate the PN5 maintained the stability in the presence of the enzyme for the physiological function. AMPs contribute only minimally the development of bacterial resistance (43). Very-low-level resistance results from mutation and gene amplification of AMPs, and antibiotic-resistant bacteria rarely exhibit cross-resistance to AMPs (44). We induced resistance to PN5, ceftazidime, and meropenem in the E. coli ATCC 25922 strain. As predicted, PN5 retained its activity even at passage 25 and did not induce resistance. This result suggests that PN5 induces less resistance compared to antibiotics (Fig. 5).

AMPs interact with bacterial cell wall components, and Gram-negative bacteria contain LPS in the outer membrane (39). LPS is an endotoxin derived from the outer layer of Gram-negative bacteria (18), and innate immune response is triggered by LPS. AMPs neutralize LPS or dissociate LPS aggregates, thereby, blocking its harmful effects (45). To investigate the anti-inflammatory effect of PN5, we examined whether PN5 interacts with LPS. The results showed that PN5 was combined with LPS with an α-helical structure, suggesting that PN5 has anti-inflammatory effects. PN5 affected the expression of inflammatory cytokines produced in RAW 264.7 cells stimulated with E. coli LPS to produce proinflammatory cytokines such as TNF-α and IL-6. TLR4 is responsible for activating NF-κB signaling and the innate immune system against pathogens as a member of the pattern recognition receptor family (46). In cytokine and receptor expression analysis, PN5 decreased the proinflammatory cytokines TLR4 and TNF-α in RAW264.7 cells stimulated with LPS, depending on the concentration. PN5 neutralized immune stimulation by LPS by downregulating TLR4 and TNF-α. Activation of these proinflammatory proteins promotes NF-κB and MAPK signal transduction. We focused on NF-κB and MAPK signaling, which play important roles in regulating inflammatory responses inimmune cells. Activation of NF-κB caused by invasion of pathogen induces proinflammatory cytokines and chemokines (47). MAPK, which includes subgroups of p38, ERK1/2, and JNK, belongs to the family of serine/threonine kinases (48). We found that LPS activates NF-κB and MAPK. In RAW 264.7 cells stimulated by E. coli LPS, PN5 reduced the expression levels of phosphorylated NF-κB and MAPK expression, indicating that PN5 exerts antibacterial and immunomodulatory activities to inhibit the inflammatory response.

We also examined the immunomodulatory effects of PN5 *in vivo*. LPS/d-GalN has been used as a model of septic shock that causes acute liver injury (Fig. S9A to D). The endotoxin LPS activates Kupffer cells, and d-GalN enhances the acute toxicity of LPS. The combined treatment of LPS and d-GalN causes liver-specific injury resulting in septic shock (26, 49). Survival and apoptotic liver injury in response to LPS and d-GalN has been reported to depend on TNF-α activation (50). In mice treated with LPS and d-GalN, PN5 reduced liver damage by inhibiting TNF-α, IL-6, and IL-1β mRNA expression. In the development of sepsis, hepatic dysfunction occurs, and systemic inflammation with liver failure is caused by high endotoxin levels (51). Histologically, inflammatory symptoms in the liver caused by LPS and d-GalN were observed along with hemorrhage and infiltration of immune cells. The symptoms were reduced by PN5 administration. Thus, PN5 exhibited anti-inflammatory effects in mice stimulated with E. coli LPS and sensitized with d-GalN.

In conclusion, we identified that PN5 can be an alternative to antibiotics. PN5 exhibits antimicrobial activity against MDR E. coli strains. PN5 did not exhibit toxicity against mammalian cells. Moreover, PN5 did not induce resistance to E. coli ATCC 25922. PN5 formed pores in the bacterial membrane by a toroidal-pore model (Fig. S10A). Moreover, PN5 is stable against proteolytic degradation and can neutralize LPS. PN5 exerted anti-inflammatory effects by mediating NF-κB and MAPK signaling in RAW264.7 cells stimulated with E. coli LPS. *In vivo*, inflammatory responses induced by LPS and d-GalN were inhibited by PN5 treatment. Based on these observations, PN5 has antimicrobial activity against MDR E. coli strains and is an anti-inflammatory agent capable of immunomodulation of the inflammatory response caused by LPS (Fig. S10B). Thus, PN5 is a promising therapeutic agent for sepsis or bacterial infection without potential for resistance development. Furthermore, current studies address the combined administration of antibiotics and AMPs as a novel approach to fight bacterial resistance (52). *In vivo* evaluation of the synergistic effect of PN5 and antibiotics is recommended in future studies.

## MATERIALS AND METHODS

### Materials.

Dimethyl sulfoxide, 3-(4,5-dimethylthiazol-2-yl)-2,5-diphenyltetrazolium bromide (MTT), NPN, ONPG, 3,3′-dipropylthiacarbocyanine iodide [DiSC_3_(5)], 2,2,2-trifluoroethanol, calcein, fluorescein isothiocyanate-dextrans (FD 4, 10, and 20), E. coli O111:B4 LPS, elastase (324681), S. aureus LTA (L2525), and d-(+)-galactosamine hydrochloride were obtained from Sigma-Aldrich (St. Louis, MO, USA). l-α-Phosphatidylethanolamine (PE [from E. coli]), l-α-phosphatidylglycerol (PG) (from E. coli), l-α-phosphatidylcholine (PC) (from chicken egg), cholesterol (CH [from ovine wool]), and sphingomyelin (SM [from porcine brain]) were obtained from Avanti Polar Lipids (Alabaster, AL). SYTOX green and carboxytetramethyl rhodamine succinimidyl ester were obtained from Molecular Probes (Eugene, OR, USA). SDS was obtained from AMRESCO (Solon, OH, USA). TNF-α ELISA kits were purchased from R&D Systems (Minneapolis, MN, USA). TNF-α (PA140281; Invitrogen), IL-6 (PM626; Invitrogen), NF-κB (PA516545; Invitrogen), IκBα (MA5-15132; Invitrogen), phospho-IκBα (MA5-15087; Invitrogen), p38 (AHO1202; Invitrogen), phosphor-p38 (MA515182; Invitrogen), ERK (136200; Invitrogen), JNK (AHO1362; Invitrogen), and phospho-JNK (700031; Invitrogen) were obtained from Thermo Fisher Scientific (Waltham, MA, USA). Phosphor-ERK (no. 4370) was purchased from Cell Signaling Technology (Danvers, MA, USA). TLR4 (sc-293072), phospho-NF-κB (sc-136548), β-actin (sc-47778), mouse IgGκ light-chain binding protein conjugated to horseradish peroxidase (sc-516102), and mouse anti-rabbit IgG-horseradish peroxidase (sc-2357) were purchased from Santa Cruz Biotechnology (Dallas, TX, USA).

### Microbial strains.

E. coli (ATCC 25922), P. aeruginosa (ATCC 27853), and S. aureus (ATCC 25923) were obtained from the American Type Culture Collection (ATCC) (Manassas, VA, USA). *S.* Typhimurium (KCTC 1926), A. baumannii (KCTC 2508), B. subtilis (KCTC 2217), and L. monocytogenes (KCTC 3710) were obtained from the Korea Collection for Type Cultures (KCTC) (Jeollabuk-do, South Korea). All MDR E. coli strains (ASEC 1 to 19) were obtained from Asan Hospital (South Korea).

### Peptide synthesis and preparation.

Peptides were synthesized using the 9-fluorenylmethoxycarbonyl (Fmoc) solid-phase method on Rink amide 4-methylbenzhydrylamine resin using a Liberty microwave peptide synthesizer (CEM Co., Matthews, NC, USA) (53). Peptides bound to the resin were treated with 20% piperidine in dimethylformamide to remove the Fmoc protection groups from the N-terminal amino acids. For rhodamine labeling, resin-bound peptides were reacted with rhodamine-SE in dimethylformamide containing 5% (vol/vol) diisopropylethylamine. After being gently mixed for 24 h in the dark, the resins were washed with dimethylformamide and then with dichloromethane. The peptides were cleaved from their respective resins, precipitated with ether, and extracted. The crude peptides were purified by RP-HPLC (Shimadzu, Kyoto, Japan) on a Jupiter C_18_ column (4.6 by 250 mm, 300 Å, 5 μm) with a 10 to 60% acetonitrile gradient in water containing 0.05% trifluoroacetic acid. Peptide purity (99.7%) was then determined by analytical RP-HPLC on a Jupiter C_18_ column (4.6 by 250 mm, 90 Å, 4 μm). The molecular mass of the peptides was confirmed by matrix-assisted laser desorption ionization–mass spectrometry (Kratos Analytical, Inc., Chestnut Ridge, NY, USA).

### CD spectroscopy for secondary structure analysis.

CD spectroscopy of the peptides was performed at room temperature (25°C) using a Jasco 810 spectropolarimeter (Tokyo, Japan) in a 1-mm-path-length cell. The temperature was regulated by a PTC-423S controller set at 20°C. CD spectroscopy spectra were recorded at wavelengths of 190 to 250 nm at a peptide concentration of 50 μM in a mixture of 10 mM sodium phosphate buffer (pH 7.4, aqueous environment), 10 mM and 30 mM SDS (Sigma-Aldrich), and 30% and 50% TFE (Sigma-Aldrich). The peptide concentration was set at 50 μM. For each spectrum, the data from four scans were averaged and smoothed using the J720/98 system program (version 120C). CD data were expressed as the mean residue ellipticity [θ] MRW in degrees square centimeter per decimole (54).

### MIC of AMPs against microorganisms.

Bacterial cells were cultured at 37°C in the appropriate culture media with shaking (180 rpm). Luria-Bertani broth (LB) (244620; BD Difco) was used for E. coli ATCC 25922, ASEC strains, and B. subtilis KCTC 2217. Nutrient broth (NB) (234000; BD Difco) was used for P. aeruginosa ATCC 27853, *S.* Typhimurium KCTC 1926, and A. baumannii KCTC 2508. Tryptic soy broth (TSB) (211825; BD Difco) was used for S. aureus ATCC 25923. Finally, brain heart infusion (BHI) (237500; BD Difco) was used for L. monocytogenes KCTC 3710. The antimicrobial activity of each peptide was determined using microdilution assays. To this end, 2-fold serial dilution series of each peptide (from 1 to 64 μM) were added to duplicate media with 10 mM sodium phosphate buffer (pH 7.4). Fifty microliters of the peptide and antibiotic solution was added to each well of a 96-well plate (32096, surface nontreatment; SPL Life Sciences). Then, 50 μL of 2 × 10^5^ CFU mL^−1^ of bacteria was added to each well, and the samples were incubated for 18 to 24 h without shaking at 37°C.

### Cell culture, viability, and hemolytic activity.

RAW 264.7 cells (mouse macrophage cells) were maintained in high-glucose Dulbecco’s modified Eagle medium (DMEM) (Welgene, Daegu, South Korea) supplemented with 10% fetal bovine serum (FBS) (Gibco) and 1% penicillin at 37°C in humidified 5% CO_2_. Cells (2 × 10^5^ cells well^−1^) were seeded into DMEM supplemented with 10% FBS medium in a 96-well plate. The MTT assay was performed after culturing the cells in 96-well plates at a density of 2 × 10^5^ cells well^−1^, followed by treatment with serially diluted PN5, magainin 2, and melittin. After 24 h, the medium was discarded, and 100 μL DMEM and 10% FBS medium containing 0.5 mg mL^−1^ MTT were added to each well. After 4 h of incubation, the medium was discarded and dimethyl sulfoxide was added to each well to solubilize the formazan. The optical densities at 570 and 650 nm (OD_570_ and OD_650_, respectively) of the treated cells were compared to those of control cells. The hemolytic activity of the peptides was evaluated as the release of hemoglobin from an 8% mRBC suspension. A 100-μL aliquot of the 8% mRBC suspension was added to the wells of a 96-well plate, and 100 μL of peptide solution in phosphate-buffered saline (PBS) was added at a final peptide concentration of 200 μM. After incubation for 1 h at 37°C with shaking (50 rpm), the suspensions were centrifuged at 1,000 × *g* for 5 min. The absorbance of the supernatant was measured at 414 nm using a Versa Max microplate reader (Molecular Devices). As a control, no hemolysis and complete hemolysis were determined in PBS and 0.1% Triton X-100, respectively. The degree of hemolysis was calculated using the following equation as described in reference 55:

$$\%\;\mathrm{hemolysis}=[(A_{414}\;\mathrm{with}\;\mathrm{peptide}-A_{414}\;\mathrm{with}\;\mathrm{PBS})/(A_{414}\;\mathrm{with}\;0.1\%\;\text{Triton}\;\text{X-100}-A_{414}\text{ with PBS)] }\times \text{ 100 }$$

### Time-dependent cytotoxicity assay.

An IncuCyte cytotoxicity assay (Essen BioScience, Inc., Ann Arbor, MI, USA) was performed to evaluate time-dependent cytotoxicity. RAW 264.7 cells (2 × 10^5^ cells well^−1^) were seeded into 96-well culture plates and then treated with peptides and IncuCyte Cytotox green reagent (Essen BioScience, Inc.). Up to 200 μM PN5 and 25 μM melittin were used to treat RAW 264.7 cells, with the concentrations serially diluted by half. Cell viability was observed for 48 h at 37°C in a humidified atmosphere of 5% CO_2_. The cells were stained with IncuCyte Cytotox green reagent. The viability was measured using IncuCyte software (Essen BioScience, Inc.) (56).

### Time-kill kinetic assay.

E. coli 25922 and S. aureus 25923 (2 × 10^5^ CFU mL^−1^) were treated with 1× and 2× MIC of PN5. At different time points, aliquots of this mixture were serially diluted with LB and TSB media and then plated onto agar plates. The cells were incubated overnight at 37°C, and bacterial colonies were counted.

### Biofilm formation and effect of AMPs on inhibition of biofilm.

Crystal violet staining was performed to determine the degree of biofilm formation by the E. coli 25922, S. aureus 25923, and ASEC strains. E. coli 25922 and S. aureus 25923 were cultured overnight in LB and TSB media with aeration at 180 rpm, respectively. Bacterial suspensions with 0.2% glucose (5 × 10^5^ CFU mL^−1^) were added to a 96-well plate (30096, surface treatment; SPL Life Sciences) and incubated for 24 h at 37°C. The supernatant was removed and fixed with 100% methanol. Next, 100 μL of crystal violet was added to the plate and incubated to dye the cells for 1 h, followed by rinsing the cells three times with distilled water (dH_2_O). After the crystal violet was dissolved with 95% ethanol, the mass of biofilm formation at 595 nm was measured using a Versa Max microplate reader (Molecular Devices). The inhibitory effect of PN5 against biofilm formed by E. coli 25922, S. aureus 25923, and three MDR E. coli strains (ASEC 2, 16, and 18) were determined. These cells were cultured overnight in LB and TSB media. Bacterial suspensions (5 × 10^5^ CFU mL^−1^) containing 0.2% glucose were added to 96-well plates and PN5 was serially diluted to a final concentration of 32 μM. The minimal biofilm inhibitory concentration (MBIC) was determined in three independent experiments. The percentage of biofilm formation was measured using the equation (*A*_595_ of treated biofilm/*A*_595_ of untreated biofilm) × 100. Similarly, the MDR ASEC 1 to 19 strains were cultured overnight in LB medium and seeded into 96-well plates with medium containing 0.2% glucose at 5 × 10^5^ CFU mL^−1^ of the bacterial suspension. Biofilm staining was performed as described above. SYTO 9 (6 μM) fluorescence in LIVE/DEAD BacLight was used to visualize the degree of biofilm formation. PN5 (16, 8, 16, and 16 μM) and antibiotics (32 μM) were added to 5 × 10^5^ CFU mL^−1^ of E. coli 25922, ASEC 2, ASEC 16, and ASEC 18. After 24 h, the culture supernatant of the peptide and bacteria was discarded, and the pellet was fixed with 100% methanol. A fluorescence mixture of SYTO 9 and propidium iodide was then added to each well, and the fluorescence was evaluated with an EVOS FL color imaging system (Thermo Fisher Scientific) with a 4× objective (22).

### NPN uptake assay.

The ability of PN5 to permeate the outer bacterial membrane was determined based on the uptake of NPN, a fluorescent dye. Briefly, E. coli 25922 cells grown to the logarithmic phase at 37°C were harvested by centrifugation at 6,000 × *g* and 4°C for 10 min. The bacterial cells were washed twice with 5 mM HEPES buffer (pH 7.4) and resuspended to OD_600_ of 0.4. NPN was added to the bacterial suspension at a final concentration of 10 μM. Aliquots of the peptides were added to the bacterial suspensions. Background fluorescence was recorded using a fluorescence spectrophotometer. The excitation and emission wavelengths were set to 350 and 420 nm, respectively. The values were converted to percentage of NPN uptake using the equation % NPN uptake = [(F_obs_ − F_0_)/(F_100_ − F_0_)] × 100, where F_obs_ is the fluorescence observed at each peptide concentration, F_0_ is the initial fluorescence of NPN with E. coli in the absence of peptides, and F_100_ is the fluorescence of NPN upon the addition of 0.1% Triton X-100, which was used as a positive control (57).

### ONPG assay and cytoplasmic membrane depolarization assay.

The ONPG assay was performed to assess the inner membrane permeability of E. coli 25922. Peptides at 0.5×, 1×, 2×, and 4× MIC were added along with a final ONPG concentration of 1.5 mM. The E. coli 25922 cells were washed twice in sodium phosphate buffer (pH 7.4) and adjusted to an OD_600_ of 0.4. ONPG is cleaved by β-galactosidase in E. coli 25922 to release *o*-nitrophenol at 420 nm. Time-dependent fluorescence was measured in a 96-well plate at 37°C using a Versa Max microplate reader (44). Cytoplasmic membrane depolarization was determined using the fluorescent dye DiSC_3_(5). Exponential-phase bacteria at an OD_600_ of ~0.05 in 5 mM HEPES and 20 mM glucose buffer (pH 7.4) were incubated with 2 μM DiSC_3_(5). The suspension was incubated for 30 min to stabilize the fluorescence intensity. Next, 100 mM KCl was added to equilibrate the cytoplasmic and external K^+^ concentrations, followed by addition of bacterial suspensions at each peptide concentration. The fluorescence intensity was measured using 96-well black plates at an excitation wavelength of 622 nm and emission wavelength of 670 nm (58).

### SYTOX green uptake assay.

SYTOX green (Molecular Probes) is a high-affinity nucleic acid stain. E. coli 25922 and S. aureus 25923 were cultured in LB and TSB media overnight at 37°C with shaking (180 rpm). The bacteria were then washed twice with 10 mM sodium phosphate buffer (pH 7.4) and suspended at an OD_600_ of 0.4. The bacterial suspension was incubated with 1 μM SYTOX green for 15 min in the dark. After adding PN5 at final concentrations of 0.5×, 1×, 2×, and 4× MIC, the uptake of SYTOX green was determined at excitation and emission wavelengths of 485 and 520 nm, respectively, every 5 min for 1 h using a fluorescence spectrophotometer (49).

### Confocal laser scanning microscopy for observation of rhodamine-labeled PN5.

Carboxytetramethyl rhodamine succinimidyl ester-labeled PN5 was diluted in 10 mM sodium phosphate buffer (pH 7.4) and stored at −20°C. E. coli 25922 and S. aureus 25923 were washed twice with sodium phosphate buffer (pH 7.4) and suspended at an OD_600_ of 0.4. The washed E. coli 25922 and S. aureus 25923 were treated with 2× MIC of rhodamine-labeled PN5 for 30 min with shaking (180 rpm). Fluorescence spectra were measured at 5-min intervals. Rhodamine-labeled PN5 was added to E. coli 25922 and S. aureus 25923 as described above for fluorescence spectrophotometry. The rhodamine-labeled PN5 at 2× MIC was added to the individual bacterial suspensions. After incubation for 30 min with shaking (180 rpm), the bacterial pellet was centrifuged at 6,000 × *g* for 5 min and washed three times with PBS. The localization of the rhodamine-labeled PN5 was observed under an inverted LSM510 laser scanning microscope (Carl Zeiss, Gottingen, Germany) equipped with a helium/neon laser (543 nm).

### Liposome preparation and calcein leakage.

The lipid compositions were prepared at different molar ratios of PE to PG (7:3 [wt/wt]) and PC to CH to SM (1:1:1 [wt/wt]) and dispensed with chloroform in a glass tube. The mixture was dried using argon gas until the smell of chloroform disappeared and then lyophilized for over 2 h or overnight and suspended in 2.6 mL PBS. The mixture was vortexed for 30 min until the mixture in PBS became milky. Freezing and thawing cycles were performed approximately nine times. Freezing was carried out using nitrogen, and thawing was carried out in a water bath. The samples were loaded into one of the gas-tight syringes and carefully placed into one end of the Avanti Mini-Extruder (Avanti Polar Lipids). The filled syringe was gently pushed until the lipid solutions were completely transferred to an alternate syringe. PE-PG (7:3 [wt/wt]) and PC-CH-SM (1:1:1 [wt/wt]) liposomes were stored at 4°C (48). Membrane activity was measured as the leakage of calcein encapsulated in the liposomes by the peptide. PE-PG (7:3 [wt/wt]) and PC-CH-SM (1:1:1 [wt/wt]) were mixed with 70 mM calcein in a glass tube and lyophilized. The lipids were suspended in 2.6 mL PBS buffer and vortexed. Lipid suspensions were frozen and thawed to form liposomes and passed through a Sephadex G-50 column (Amersham Biosciences). Liposomes encapsulating calcein were separated in the column. Untrapped calcein was removed by gel filtration on a Sephadex G-50 column. The suspension was then extruded 30 times using polycarbonate membranes (200-nm pore size; Avanti Polar Lipids). In the calcein release assay, the molar ratios of PN5 and 10 μM liposomes were 0.025, 0.05, 0.1, 0.4, 0.8, and 1.2. Real-time fluorescence intensity was monitored at excitation and emission wavelengths of 480 and 520 nm, respectively (59).

### Intracellular influx of FD.

FD 4, 10, and 20 (Sigma-Aldrich) were used as model cytoplasmic components. FD 4 (3.9 kDa; diameter, ~2.8 nm), FD 10 (9.9 kDa; diameter, ~4.6 nm), and FD 20 (19.8 kDa; diameter, ~6.6 nm) were used to determine the membrane pore size based on their ability to permeate the membrane. After peptide treatment, fluorescence imaging was performed to investigate the influx of FD against E. coli ATCC 25922. FD 4, 10, and 20 were added to E. coli ATCC 25922 at final concentrations of 0.32, 0.16, and 0.16 mg mL^−1^, respectively. E. coli ATCC 25922 was washed twice with PBS and adjusted to an OD_600_ of 0.4. A concentration of 2× MIC of peptides was added to E. coli ATCC 25922 cells treated with FD. After incubation for 30 min, fluorescence imaging was performed using the EVOS FL color imaging system with a 60× objective (39).

### Resistance development.

E. coli ATCC 25922 cells were incubated overnight at 37°C in LB medium with shaking (180 rpm) and then adjusted to 2 × 10^5^ CFU mL^−1^; 0.5× MIC of PN5 and antibiotics were added to the medium and cultured overnight at 37°C with aeration (180 rpm). The MIC was determined by measuring the OD_600_ using a Versa Max microplate reader. On the next day, to measure the MIC with the bacteria grown after treatment with 0.5× MIC, the bacteria and antibiotic were adjusted to the same concentration, and the bacteria were cultured by adding 0.5× MIC in the same manner. The initial culture was continuously cultured for 25 passages in this manner.

### Resistance to proteolytic degradation by elastase.

The stability of PN5 in the presence of the enzyme was measured by treatment with elastase. First, 10 μg mL^−1^ elastase in 0.1 M Tris-HCl (pH 8.0) was incubated with 50 μg PN5 at 37°C with shaking (180 rpm), with aliquots collected at 0, 30, 60, 120, 360, 540, and 720 min. After incubation, each sample was observed by RP-HPLC with a Jupiter C_18_ column (4.6 by 250 mm, 300 Å, 5 μm). E. coli 25922 and S. aureus 25923 were cultured in LB and TSB media with shaking (180 rpm) at 37°C, washed twice with 10 mM sodium phosphate buffer (pH 7.4), suspended in 10 mL LB and TSB media at 2 × 10^5^ CFU mL^−1^, mixed with 0.2% agarose, and dried for 1 h. Paper disks (6 mm) were placed on the dried agarose, and 50 μg PN5 aliquots was dropped onto the disks. The disks were stored in a 37°C incubator for 24 h. PN5- and elastase (10 μg mL^−1^)-treated PN5 was prepared to evaluate the susceptibility to E. coli 25922 and S. aureus 25923. PN5 (6.25, 12.5, 25, 50, and 100 μg) was diluted in 10 mM sodium phosphate buffer (pH 7.4) and 10 μg mL^−1^ elastase. E. coli 25922 and S. aureus 25923 were prepared at 2 × 10^5^ CFU mL^−1^. Peptides with 10 μg mL^−1^ elastase and bacteria were incubated for 9 h with shaking (180 rpm) and observed using a visible spectrophotometer (Pharmacia Biotech, Uppsala, Sweden) (60).

### LPS and LTA binding assay.

The LPS and LTA binding assay was performed to investigate the interaction between PN5 and E. coli O111:B4 LPS and S. aureus LTA (Sigma-Aldrich) by CD spectroscopy. E. coli LPS and S. aureus LTA were diluted in 10 mM sodium phosphate buffer (pH 7.4) to 1 mg mL^−1^. The peptide (50 μM) was added to E. coli LPS and S. aureus LTA. The temperature was regulated by a PTC-423S controller and was set to 20°C. The spectra were measured from 190 to 250 nm using a CD spectropolarimeter (Jasco) operating at room temperature (25°C) (61).

### ELISA.

The expression levels of proinflammatory cytokines were evaluated using a mouse TNF-α ELISA kit. E. coli LPS (0.1 μg mL^−1^) was pretreated for 30 min, and 2, 4, and 8 μM PN5 were added to RAW 264.7 cells seeded at 5 × 10^5^ cells well^−1^. After incubation for 24 h, the supernatants were collected. TNF-α secretion was measured in the culture supernatant using the mouse TNF-α ELISA kit according to the manufacturer's protocols. The absorbance values for TNF-α were determined at 450 nm using a Versa max microplate reader.

### Immunofluorescence imaging.

To evaluate whether PN5 regulates the expression of TNF-α, 5 × 10^5^ RAW 264.7 cells were seeded into each well of 6-well plates. After incubation for 24 h at 37°C, the cells were fixed with 4% paraformaldehyde (pH 7.4) for 10 min and washed three times with PBS. The cells were then permeabilized with 0.1% Triton X-100 and blocked with 2% bovine serum albumin for 1 h at room temperature. RAW 264.7 cells were incubated with primary antibody in 0.1% bovine serum albumin overnight at 4°C. After washing, FITC-conjugated goat anti-rabbit secondary antibody diluted in 0.1% bovine serum albumin was added to the RAW 264.7 cells for 1 h at room temperature. The cells were washed three times and then observed using an EVOS imaging system with a 40× objective.

### Western blot analysis.

RAW 264.7 cells seeded at 5 × 10^5^ cells per well were subcultured in DMEM (Welgene, Daegu, South Korea) and 10% FBS (Gibco). The cells were pretreated with 0.1 μg mL^−1^ E. coli LPS for 30 min and then incubated with 2, 4, and 8 μM PN5 for 24 h. Next, the cells were washed twice with ice-cold PBS and lysed with protein extraction solution (iNtRON Biotechnology, Gyeonggi-do, South Korea). Protein in the cell lysates was quantified using the Bradford protein assay (Bio-Rad, Hercules, CA, USA). The centrifuged supernatant was collected and stored at −70°C. The quantified proteins were resolved by SDS-PAGE. After separation, the protein was transferred to a polyvinylidene fluoride membrane (0.45-μm pore size; Merck Millipore). The membrane was blocked with 5% skim milk in TBST buffer (20 mM Tris-HCl, 150 mM NaCl, 0.1% Tween 20 [pH 7.6]) and then reacted with the primary antibody at 4°C overnight. The primary antibodies used are as follows: TLR4 (1:200) (sc-293072; Santa Cruz Biotechnology), TNF-α (1:1,000) (PA140281; Invitrogen), IL-6 (1:200) (PM626; Invitrogen), NF-κB (1:1,000) (PA516545; Invitrogen), phospho-NF-κB (1:1,000) (sc-136548; Santa Cruz Biotechnology), IκBα (1:1,000) (MA5-15132; Invitrogen), phospho-IκBα (1:1,000) (MA5-15087; Invitrogen), p38 (1:1,000) (AHO1202; Invitrogen), phosphor-p38 (1:1,000) (MA515182; Invitrogen), ERK (1:1,0000) (136200; Invitrogen), phosphor-ERK (1:1,000) (#4370; Cell Signaling Technology), JNK (1:1,000) (AHO1362; Invitrogen), phospho-JNK (1:1,000) (700031; Invitrogen), and β-actin (1:5,000) (sc-47778; Santa Cruz Biotechnology). After several washes with TBST buffer, the membranes were incubated with the secondary antibody (mouse IgG-κ light-chain binding protein conjugated with horseradish peroxidase (1:10,000) (sc-516102) and mouse anti-rabbit IgG-horseradish peroxidase (1:10,000) (sc-2357) at room temperature for 2 h. The membranes were washed with TBST buffer, and horseradish peroxidase-linked secondary antibody was detected using enhanced chemiluminescence (Ab Frontier, Seoul, South Korea) solution in a dark room. The detected protein bands were quantified using ImageJ software (NIH, Bethesda, MD, USA).

### *In vivo* test and survival challenge.

Male C57BL/6J mice were stabilized for 7 days. C57BL/6J mice (8 to 10 weeks old) were injected intraperitoneally with 16 μg kg^−1^ E. coli LPS and 500 mg kg^−1^ d-GalN. After 30 min, 1 and 5 mg kg^−1^ PN5 were injected into the peritoneum. All mice were observed over time and were counted to confirm the mortality of the mice at 48 h. The stabilized male C57BL/6J mice were injected with 16 μg kg^−1^ E. coli LPS and 500 mg kg^−1^ d-GalN into the peritoneum. PN5 (1 and 5 mg kg^−1^) was injected, and then the mortality of the mice was observed (62).

### Hematoxylin and eosin staining for histological analysis.

Male C57BL/6J mice (8 to 10 weeks old) were injected intraperitoneally with 16 μg kg^−1^ E. coli LPS and 500 mg kg^−1^ d-GalN; 30 min later, 1 and 5 mg kg^−1^ of PN5 were injected. After 6 h, the mice were sacrificed, and liver tissues were collected, fixed with 4% paraformaldehyde, and paraffin embedded. The tissues were sectioned and stained with hematoxylin and eosin (Merck). Histopathological changes were observed using an EVOS imaging system with 20× and 40× objectives (62).

### RT-PCR.

Total RNA was extracted from the liver of male C57BL/6J mice 6 h later using TRIzol reagent (Tri reagent; Molecular Research Center, Cincinnati, OH, USA). cDNA was generated using TOPscript RT DryMIX (Enzynomics, Daejeon, South Korea). cDNA was used for real-time quantitative PCR after being mixed with TOPreal qPCR 2× PreMIX with SYBR green (Enzynomics). Real-time PCR amplification was performed in a volume of 20 μL using a 7500 real-time PCR system (Applied Biosystems, Foster City, CA, USA). mRNA expression was normalized to that of the endogenous β-actin gene. Relative quantification was performed using the comparative threshold cycle (2^−ΔΔ^*^CT^*) method. PCR primers for mouse TNF-α, IL-6, and IL-1β were purchased from Bioneer (Daejeon, South Korea). Each PCR primer was diluted to 10 pmol and stored at −20°C.

### Statistical analysis.

Values are presented as the mean ± standard error of the mean (SEM). Statistical analyses were performed using GraphPad Prism software (version 8.0.2; GraphPad, Inc., La Jolla, CA, USA). To compare two groups, the *t* test was used. To compare three or more groups, differences among groups were evaluated by one-way analysis of variance (ANOVA) followed by a Tukey or Bonferroni multiple-comparison test. Statistical significance was set at a *P* value of *<*0.05.

### Ethical statement.

All experiments were performed in accordance with the guidelines of Chosun University, and the experiments were approved by the Ethics Committee of Chosun University (CIACUC2018-A0002-1). Informed consent was obtained from the participants of this study.
