# Supplementary material for: Therapeutic Potential of Antimicrobial Peptide PN5 against Multidrug-Resistant E. coli and Anti-Inflammatory Activity in a Septic Mouse Model
Source: Microbiol Spectr. 2022 Sep 21;10(5):e01494-22. doi: 10.1128/spectrum.01494-22 (PMC9603901; doi:10.1128/spectrum.01494-22)
Supplement: Supplemental file 1 — Download spectrum.01494-22-s0001.pdf, PDF file, 1.0 MB [file spectrum.01494-22-s0001.pdf]

Supplemental material

Therapeutic Potential of Antimicrobial Peptide PN5 against Multidrug-resistant *E. coli* and Anti-inflammatory Activity in a Septic Mouse Model

Da Dam Kang,[a] Jonggwan Park,[b] and Yoonkyung Park[a,c]

[a]Department of Biomedical Science, Chosun University, Gwangju, 61452, Republic of Korea

[b]Department of Bioinformatics, Kongju National University, Kongju 32588, Korea.

[c]Research Center for Proteinaceous Materials (RCPM), Chosun University, Gwangju, 61452, Republic of Korea.

Da Dam Kang and Jonggwan Park contributed equally to this work.

Corresponding author: Yoonkyung Park

Email; y_k_park@chosun.ac.kr

Tel: +82 62 230 6854

Table of Contents

Supplementary materials and methods S3

Supplementary Tables S5

Table S1. Estimated extent of helicity (%) of PN5 .... S5

Table S2. Antimicrobial activity of PN5 against gram-negative and positive bacteria S6

Table S3. Antimicrobial resistance information of clinically isolated multidrug-resistant *E. coli* strains from Asan hospital. S7

Table S4. Minimum inhibitory concentrations of PN5 and antibiotics against resistance-induced *E. coli* ATCC 25922. .... S8

Supplementary Figures S9

Figure S1. RP-HPLC and MALDI-TOF of PN5 .... S9

Figure S2. Biofilm formation of *S. aureus* ATCC 25923 and anti-biofilm activity of PN5 against *S. aureus* ATCC 25923 .... S10

Figure S3. Outer membrane permeability of control peptides S11

Figure S4. Permeability of control peptides on the cytoplasmic membrane S12

Figure S56. SYTOX green influx of control peptides against *E. coli* ATCC 25922 and *S. aureus* ATCC 25923 .... S13

Figure S6. The membrane permeabilization effect of PN5 against *S. aureus* ATCC 25923 .... S14

Figure S7. Anti-inflammatory effect of PN5 on *E. coli* LPS and *S. aureus* LTA S15

Figure S8. Time-dependent changes of NF-κB and TNF-α by *E. coli* LPS treatment S16

Figure S9. The lesions in the liver of mice was challenged with *E. coli* LPS and D-galactosamine (D-GalN) S17

Figure S10. Schematic diagram of anti-bacterial, anti-biofilm and anti-inflammatory mechanisms of PN5 .... S18

Reference S19

Supplementary materials and methods

Gene expression analysis of pro-inflammatory cytokines

RAW 264.7 cells (5 × 10^5 cells well^{-1}) were seeded in six-well plates and cultured in a CO_2 incubator for 24 h. They were pre-treated with 0.1 μg mL^{-1} *E. coli* LPS or *S. aureus* LTA for 30 min. Subsequently, the cells were treated with 2, 4, and 8 μM PN5 for 24 h. The RAW 264.7 cells were then isolated using TRIzol reagent. After RNA quantification, cDNA was synthesized using a TOPscript™ RT DryMIX cDNA synthesis kit. Quantitative real time-polymerase chain reaction (qRT-PCR) was performed with TOPreal qPCR 2 × premix (SYBR green) using a 7500 real-time PCR system (Applied Biosystems, Foster City, CA, USA) and primers for TNF-α, IL-6, and IL-1β genes. The following oligonucleotides were used as primers: GAPDH primers were 5′- GAA GGT GAA GGT CGG AGT CA-3′ (forward), 5′-TTG AGG TCA ATG AAG GGG TC-3′ (reverse); TNF-α primers were 5′-GGC AGG TCT ACT TTG GAG TCA TTG C-3′ (forward), 5′-ACA TTC GAG GCT CCA GTG AAT TCG G-3′ (reverse); IL-6 primers were 5′-CTG GTG ACA ACC ACG GCC TTC CCT A-3′ (forward), 5′-ATG CTT AGG CAT AAC GCA CTA GGT T-3′ (reverse); IL-1β primers were 5′-TCA TGG GAT AAC CTG CT-3′ (forward), 5′-CCC ATA CTT TAG GAA GAC ACG GGA TT-3′ (reverse).

Amplification was performed by denaturing for 10 min at 95°C. The qPCR protocol was as follows: 40 cycles of 95°C for 15 s, 60°C for 30 s, and 72°C for 30 s. For mRNA quantification, expression levels were normalized to that of GAPDH. Each sample was analyzed in triplicates.

Detection of ALT and AST in mouse serum

Ten milliliter blood tubes (BD Vacutainer, BD Biosciences) were used to collect mice blood samples. Blood was collected from mice and centrifuged at 900 ×*g* for 15 min to separate

the serum. Alanine aminotransferase (ALT) and aspartate aminotransferase (AST) were detected using a kit from Asan Pharmaceutical (Gyeonggi-do, South Korea) according to the manufacturer's instructions. The levels of AST and ALT were determined relative to a standard provided in the kit. The absorbance was measured at 505 nm using an ELISA plate reader (VersaMax, Molecular Devices, USA).

Supplementary tables

Table S1 Estimated extent of helicity (%) of PN5.

Peptide	10 mM SP buffer		30 mM SDS		50% TFE		1 mg mL^{-1} LPS		1 mg mL^{-1} LTA	
	$[\theta]_{222}$	%α-helix	$[\theta]_{222}$	%α-helix	$[\theta]_{222}$	%α-helix	$[\theta]_{222}$	%α-helix	$[\theta]_{222}$	%α-helix
PN5	195.6713	RC	-4998.34	9.99%	-4810.13	9.38%	-6239.51	14.13%	-11465.1	31.55%

% α-helix = $-([\theta]_{222}+2000)/30000 \times 100\%$, RC means random coil (1).

Table S2 Antimicrobial activity of PN5 against gram-negative and positive bacteria.

Microorganism	MIC[a] μM (μg mL^{-1})		
	PN5	Magainin 2	Melittin
**Gram (-)**			
P. aeruginosa ATCC 27853	4 (5.30)	32 (78.94)	4 (11.39)
S. Typhimurium KCTC 1926	2 (2.65)	>64 (>157.88)	4 (11.39)
A. baumannii KCTC 2508	4 (5.30)	8 (19.74)	2 (5.69)
**Gram (+)**			
S. aureus ATCC 25923	2 (2.65)	16 (39.47)	2 (5.69)
B. subtilis KCTC 2217	4 (5.30)	16 (39.47)	2 (5.69)
L. monocytogenes KCTC 3710	16 (21.22)	>64 (>157.88)	4 (11.39)

[a] Minimum inhibitory concentration (MIC) was used in three independent experiments.

Table S3 Antimicrobial resistance information of clinically isolated multidrug-resistant *E. coli* strains from Asan hospital.

	Number	Imipenem	Meropenem	Cefotaxime	Ceftazidime	Ceftriaxone	Cefoxitin	ESBL	Carbapenemase
Cabapenemase									
ASEC 1	22365	<=4 (R)	8 (R)	>32 (R)	>16 (R)	>32 (R)	>16 (R)	N	NDM-1
ASEC 2	25497	8 (R)	>8 (R)	>32 (R)	>16 (R)		>16 (R)	N	NDM-7
ASEC 3	26077	>8 (R)	>8 (R)	>32 (R)	>16 (R)		>16 (R)	N	NDM-5
ESBL									
ASEC 4	16655	>8 (R)	>8 (R)	>32 (R)	>16 (R)	>32 (R)	>16 (R)	Positive	N
ASEC 5	17113	>8 (R)	<=4 (S)	>32 (R)	>16 (R)	>32 (R)	>16 (R)	Positive	N
ASEC 6	20600	>8 (R)	>8 (R)	>32 (R)	>16 (R)	>32 (R)	>16 (R)	Positive	N
ASEC 7	25644	<=1 (S)	<=1 (S)	>32 (R)	>16 (R)		<=8 (S)	Positive	
ASEC 8	26155	<=1 (S)	<=1 (S)	>32 (R)	>16 (R)		<=8 (S)	Positive	
ASEC 9	26044	<=1 (S)	<=1 (S)	>32 (R)	>16 (R)		16 (I)	Positive	
ASEC 10	25759	<=1 (S)	<=1 (S)	>32 (R)	16 (R)		<=8 (S)	Positive	
ASEC 11	25831	<=1 (S)	<=1 (S)	>32 (R)	>16 (R)		<=8 (S)	Positive	
AmpC									
ASEC 12	16282	>8 (R)	>8 (R)	>32 (R)	16 (R)	>32 (R)	>16 (R)	N	N
ASEC 13	19100	>8 (R)	>8 (R)	>32 (R)	>16 (R)	>32 (R)	>16 (R)	N	N
ASEC 14	23739	8 (R)	8 (R)	>32 (R)	>16 (R)		>16 (R)	N	N
ASEC 15	25862	<=1 (S)	<=1 (S)	>32 (R)	>16 (R)		>16 (R)	N	
ASEC 16	25865	<=1 (S)	<=1 (S)	>32 (R)	>16 (R)		>16 (R)	N	
ASEC 17	25901	<=1 (S)	<=1 (S)	>32 (R)	>16 (R)		>16 (R)	N	
ASEC 18	25953	<=1 (S)	<=1 (S)	32 (R)	>16 (R)		>16 (R)	N	
ASEC 19	26076	<=1 (S)	<=1 (S)	>32 (R)	>16 (R)		>16 (R)	N	

*MIC measured in μg mL^{-1}, *S: susceptible,* R: resistant, *I: intermediate

*ESBL: Extended-spectrum beta-lactamases (enzymes that confer resistance to most beta-lactam antibiotics), *NDM: New Delhi metallo-beta-lactamase, *N: Negative, *Number indicates the strain storage number designated by Asan hospital

Table S4 Minimum inhibitory concentrations of PN5 and antibiotics against resistance-induced *E. coli* ATCC 25922.

**Microorganism**	**MIC (µM)**[a]		
	PN5	Ceftazidime	Meropenem
E. coli – Ceftazidime (Passage 25)	16 (16)	128 (0.25)	0.03125 (0.03125)
E. coli – Meropenem (Passage 25)	16 (16)	0.25 (0.25)	0.25 (0.03125)

[a] Minimum inhibitory concentration (MIC) was used in three independent experiments.

Supplementary figures

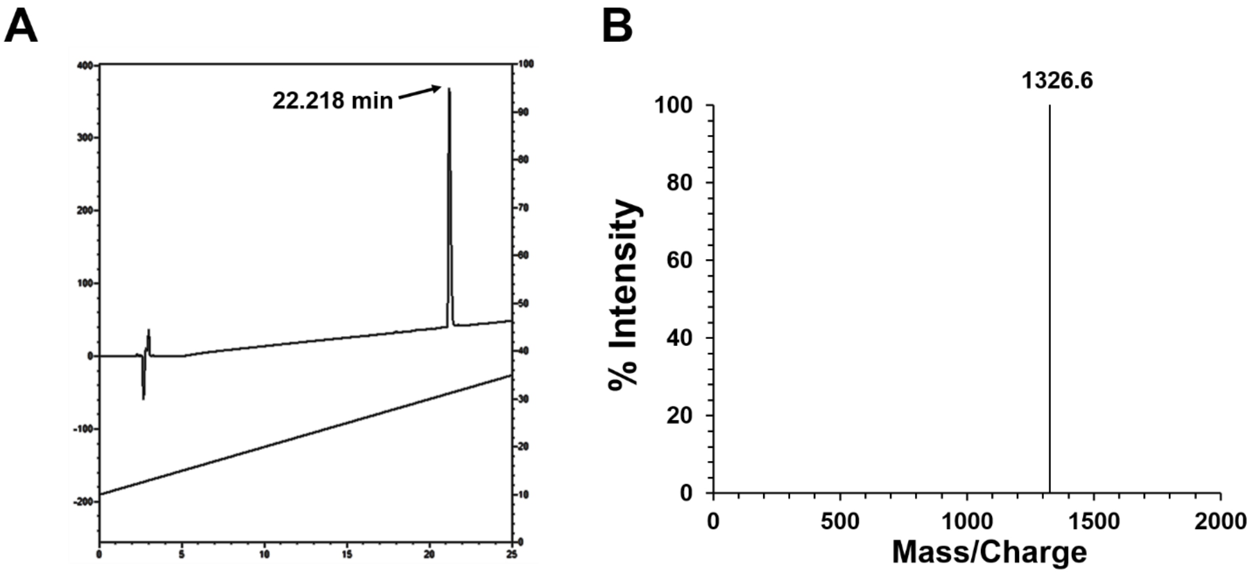

FIG S1 RP-HPLC and MALDI-TOF of PN5. (A) RP-HPLC profile on a C18 column was detected at 214 nm (99.7% purity). The arrow indicates retention time (22.218 min). (B) MALDI mass spectrometric analysis of PN5. The peak means a mass/charge value of PN5 (1326.6).

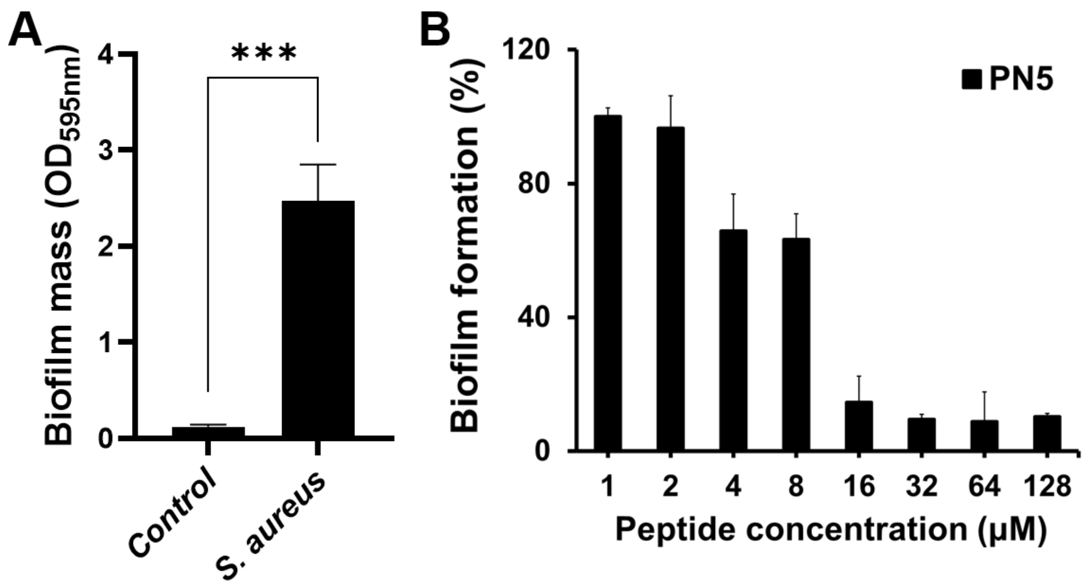

FIG S2 (A) Biofilm formation of 10 mM SP buffer-treated (negative control) and *S. aureus* ATCC 25923 in TSB with 0.2% glucose. (B) Anti-biofilm activity of PN5 on biofilm formation against *S. aureus* ATCC 25923. The biofilms stained with 0.1% crystal violet was observed at 595 nm. Values represent mean ± SEM, *** $P < 0.001$.

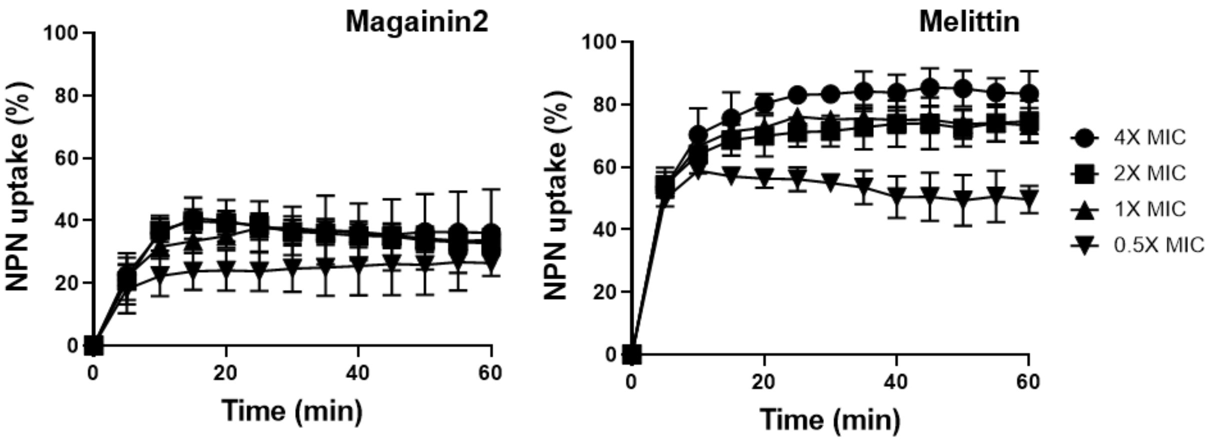

FIG S3 Outer membrane permeability of magainin 2 and melittin (control peptides). *Escherichia coli* ATCC 25922 was incubated with NPN in the presence of 0.5×, 1×, 2×, and 4× MIC of control peptides.

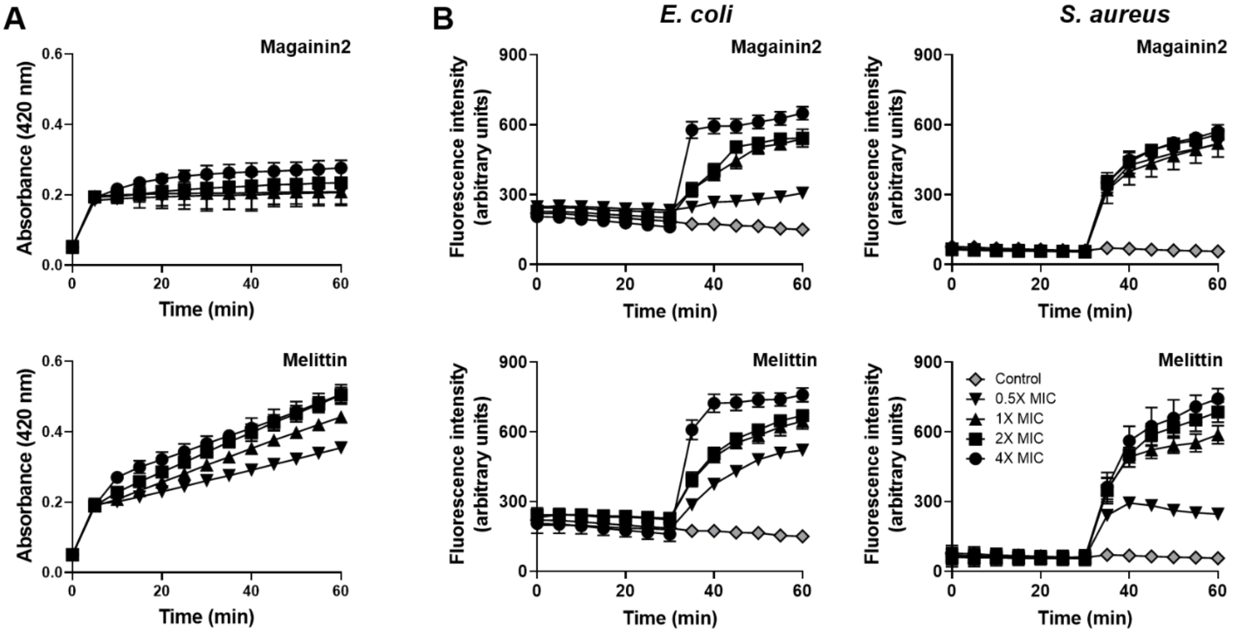

FIG S4 Permeability of control peptides on the cytoplasmic membrane. (A) Ortho-Nitrophenyl-β-galactosidase (ONPG) was cleaved by β-galactosidase of *E. coli* ATCC 25922 treated by magainin 2 and melittin at 0.5×, 1×, 2×, and 4× MIC, and hydrolysate was measured at absorbance of 420 nm (B) Cytoplasmic membrane potential of peptides with DiSC3(5). *Escherichia coli* ATCC 25922 and *S. aureus* ATCC 25923 were pre-incubated with membrane potential-sensitive dye DiSC3(5) for 100 min. Then, 0.5×, 1×, 2×, and 4× MIC of peptides were added to pre-incubated bacteria with DiSC3(5) and fluorescence changes was observed.

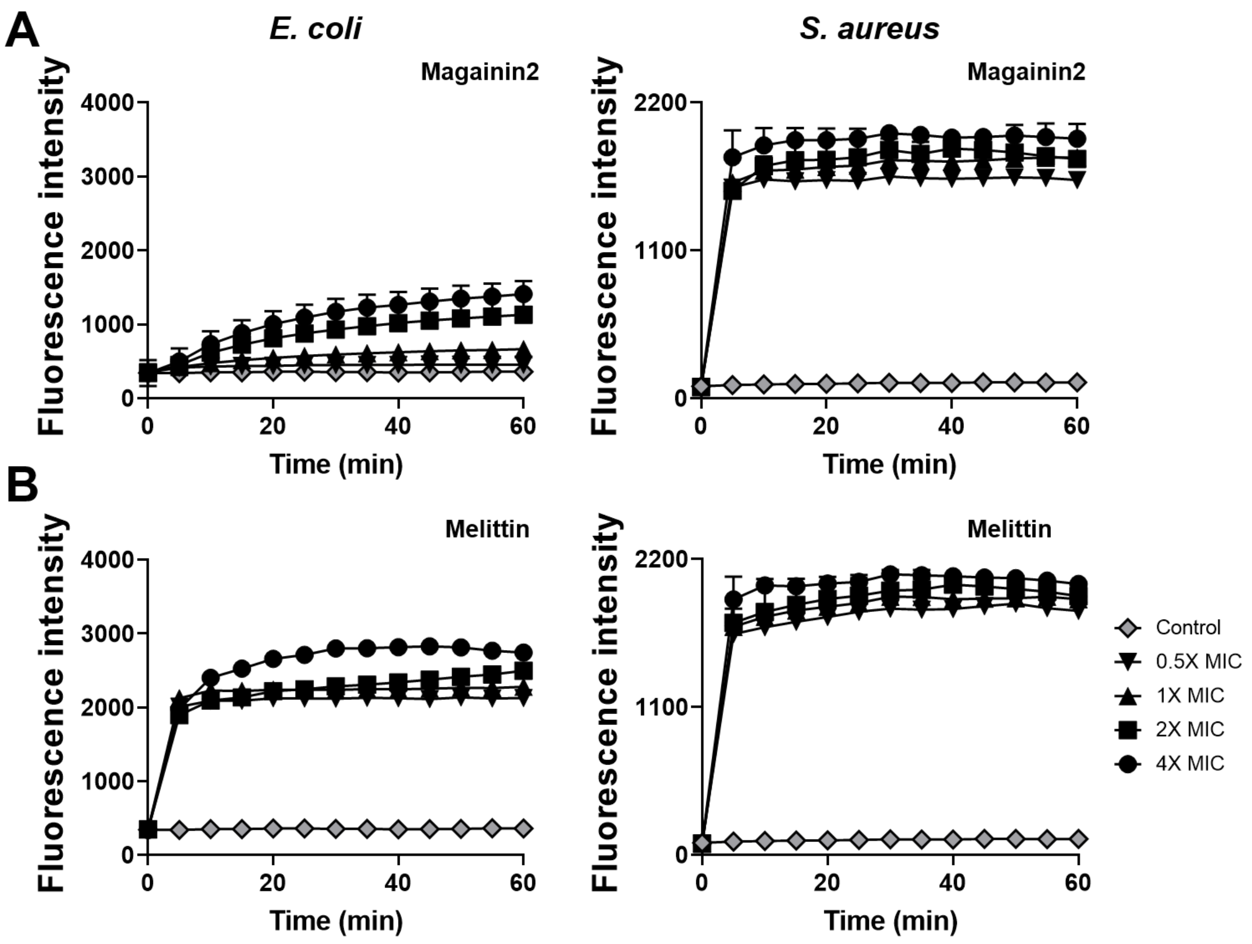

FIG S5 SYTOX green influx of control peptides against *E. coli* ATCC 25922 and *S. aureus* ATCC 25923. Additions of 0.5×, 1×, 2×, and 4× MIC of (A) magainin 2 and (B) melittin into *E. coli* and *S. aureus* to monitor the changes of fluorescence at 485 nm and 520 nm.

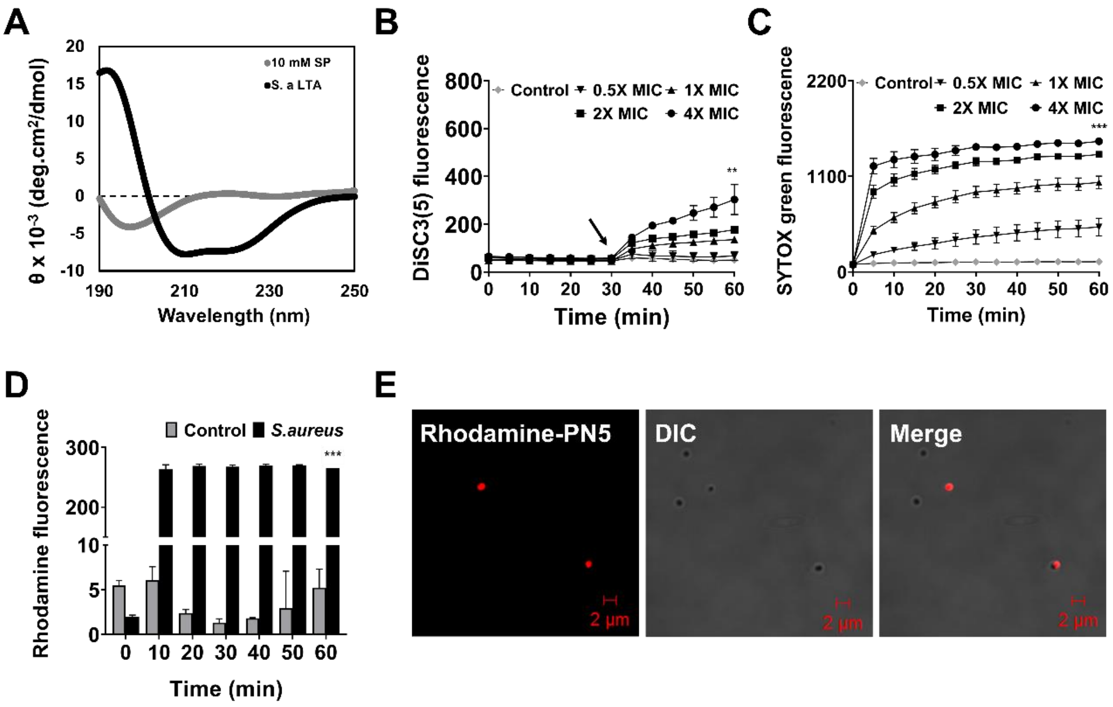

FIG S6 The membrane permeabilization effect of PN5 against *S. aureus* ATCC 25923. (A) CD analysis of the binding structure of PN5 with lipoteichoic acid (LTA). (B) Various indicated concentrations of PN5 were added at 30 min (arrow mark) to *S. aureus* ATCC 25922 with $DiSC_3$ (5). Cytoplasmic membrane potential changes were observed at 622 nm (excitation) and 670 nm (emission). (C) Fluorescence intensity of SYTOX green was measured at 485 nm (excitation) and 520 nm (emission). (D) Rhodamine-labeled PN5 was used to treat *S. aureus* ATCC 25923. PN5 labeled with rhodamine (2× MIC) was added and observed using a fluorescence spectrophotometer for 60 min. (E) Localization of rhodamine-labeled PN5 on *S. aureus* ATCC 25923 as observed using confocal laser scanning microscopy. Scale bar of *S. aureus* = 2 μm. All values represent the mean ± SEM of three individual experiments, **, $P < 0.01$ and ***, $P < 0.001$ vs control.

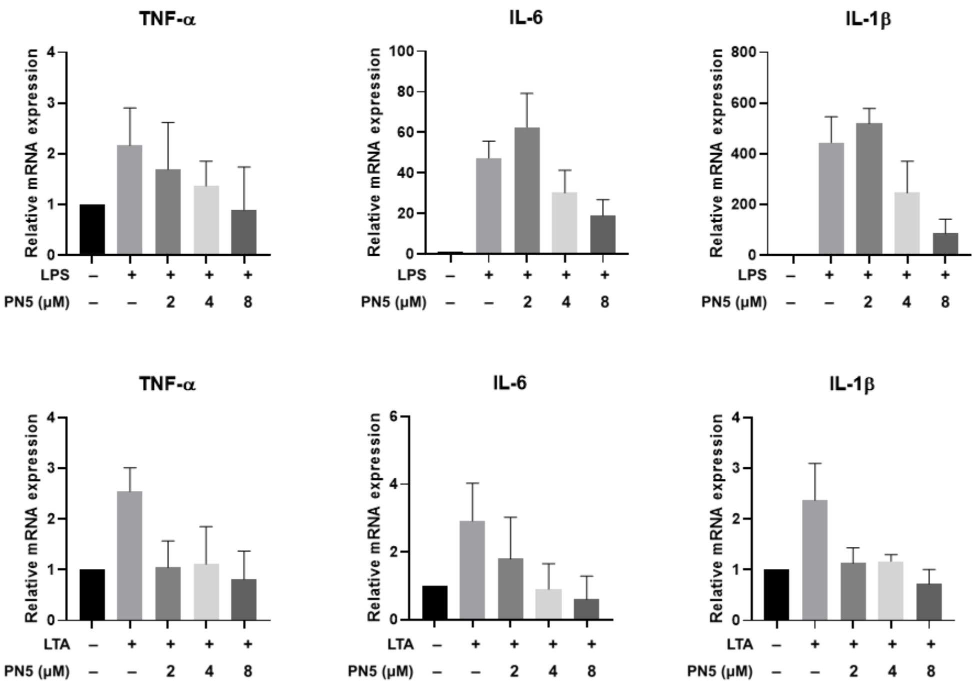

FIG S7 Anti-inflammatory effects of PN5 on *E. coli* LPS and *S. aureus* LTA. RAW 264.7 cells at 5×10^5 cells well^{-1} were pre-treated with 0.1 μg mL^{-1} *E. coli* LPS and *S. aureus* LTA for 30 min. Subsequently, 2, 4, and 8 μM of PN5 were added and incubated for 24 h. The expression of pro-inflammatory cytokines was analyzed using RT-PCR.

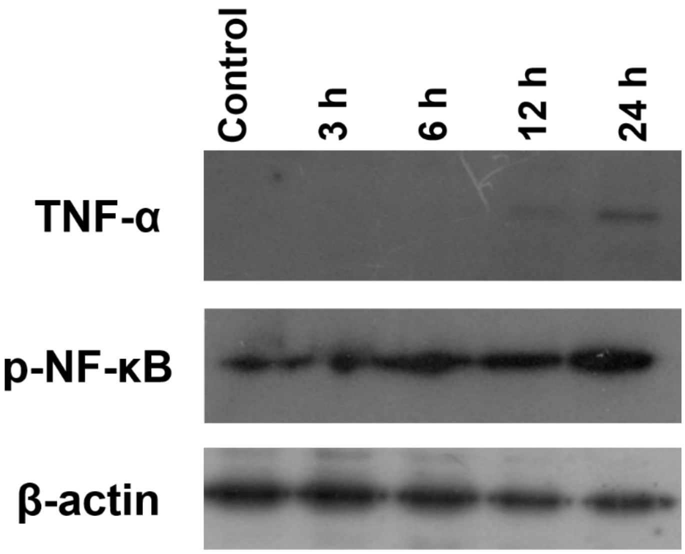

FIG S8 RAW 264.7 cells at 5×10^5 cells well^{-1} were treated with 0.1 μg mL^{-1} *E. coli* LPS for 3, 6, 12, and 24 h. The cells were harvested to extract proteins. Western blot was performed to detect the expression of TNF-α and NF-κB.

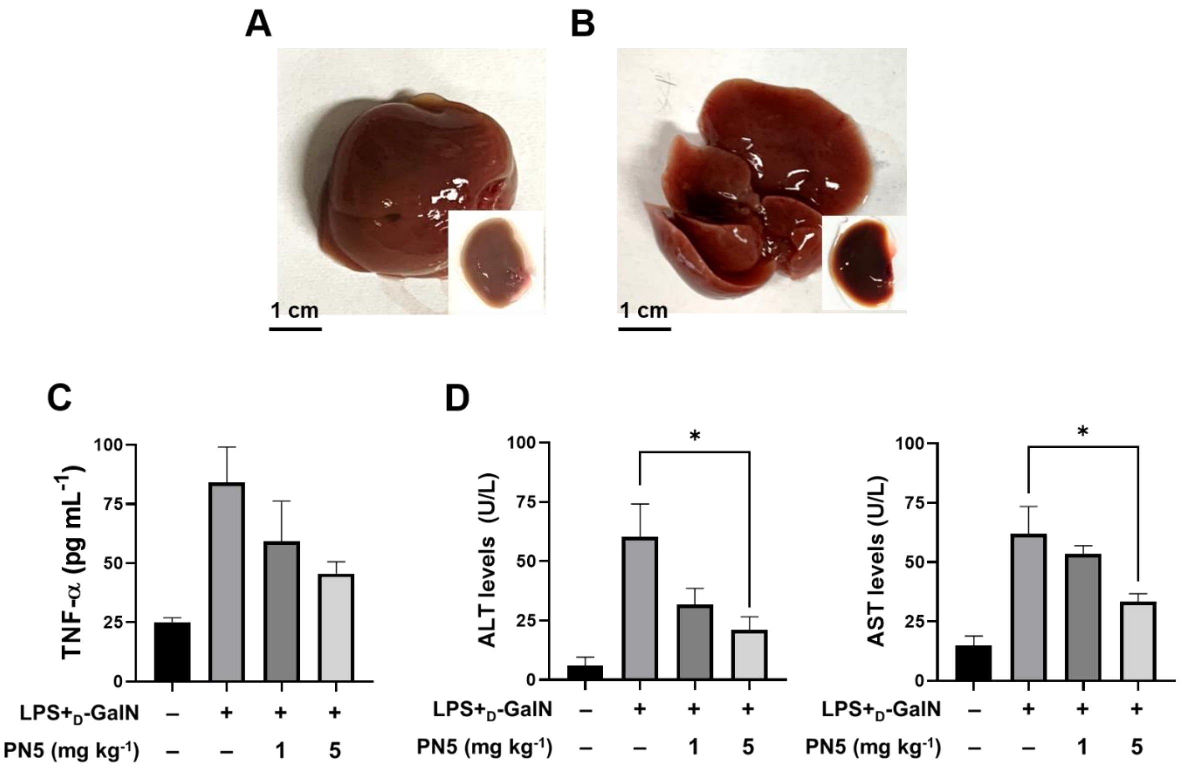

FIG S9 Lesions in the liver of mice that were treated with *E. coli* LPS and D-galactosamine (D-GalN). (A) Mice were intraperitoneally injected with PBS, and the liver was harvested. (B) Mice were intraperitoneally injected with *E. coli* LPS and D-GalN, and the liver was harvested. Scale bars = 1 cm. (C) Serum levels of TNF-α were detected using mouse ELISA kit. (D) Serum levels of ALT and AST. After treatment with *E. coli* LPS and D-GalN, PN5 (1 mg kg^{-1} and 5 mg kg^{-1}) was administered (n=5/group). After 6 h, the mouse blood samples were collected. ALT, alanine transaminase; AST, aspartate aminotransferase. Values represent the mean ± SEM. *, $P < 0.02$; by unpaired *t* test.

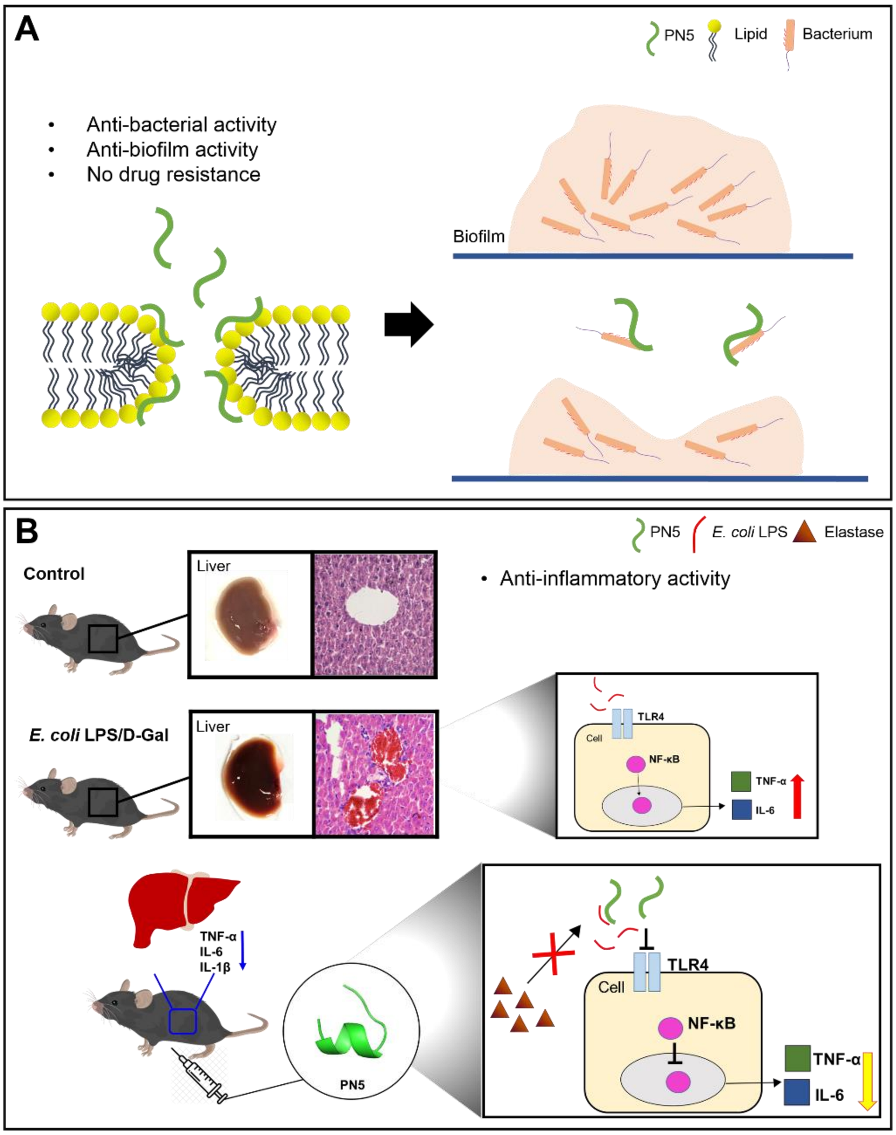

FIG S10 Schematic diagram of anti-bacterial, antibiofilm, and anti-inflammatory mechanisms of PN5.

Reference

1. Lu J, Xu H, Xia J, Ma J, Xu J, Li Y, Feng J. 2020. D- and unnatural amino acid substituted antimicrobial peptides with improved proteolytic resistance and their proteolytic degradation characteristics. Frontiers in microbiology 11:563030. https://doi.org/10.3389/fmicb.2020.563030
